# Emerging Elastic Micro-Nano Materials for Diagnosis and Treatment of Thrombosis

**DOI:** 10.34133/research.0614

**Published:** 2025-02-28

**Authors:** Chenxin Lu, Chunjian Li, Ning Gu, Fang Yang

**Affiliations:** ^1^ State Key Laboratory of Digital Medical Engineering, Jiangsu Laboratory for Biomaterials and Devices, School of Biological Science and Medical Engineering, Southeast University, Nanjing 210096, P. R. China.; ^2^ Department of Cardiology, The First Affiliated Hospital of Nanjing Medical University, Nanjing 210029, P. R. China.; ^3^ Nanjing Key Laboratory for Cardiovascular Information and Health Engineering Medicine, Institute of Clinical Medicine, Nanjing Drum Tower Hospital, Medical School, Nanjing University, Nanjing 210093, P. R. China.

## Abstract

Thrombus is a blood clot that forms in a blood vessel at the point of flaking. Thrombosis is closely associated with cardiovascular diseases caused by different sources and factors. However, the current clinical methods of thrombus diagnosis and treatment still have problems with targeting, permeability, stability, and biosafety. Therefore, in recent years, based on the development of micro/nano technology, researchers have tried to develop some new strategies for the diagnosis and treatment of thrombosis. Due to the unique structural characteristics, the micro-nano materials in physiological environments show excellent transport and delivery properties such as better in vivo circulation, longer life span, better targeting ability, and controllable cellular internalization. Especially, elasticity and stiffness are inherent mechanical properties of some well-designed micro-nano materials, which can make them better adapted to the needs of thrombosis diagnosis and treatment. Herein, this review first introduces the thrombotic microenvironment to characterize the thrombus development process. Then, to fine-tune the pathological occurrence and development of thrombosis, the role of elastic micro-nano materials for thrombus diagnosis and treatment is summarized. The properties, preparation methods, and biological fate of these materials have been discussed in detail. Following, the applications of elastic micro-nano materials in biomedical imaging, drug delivery, and therapy of thrombosis are highlighted. Last, the shortcomings and future design strategies of elastic micro-nano materials in diagnosis and treatment of clinical thrombosis are discussed. This review will provide new ideas for the use of nanotechnology in clinical diagnosis and treatment of thrombus in the future.

## Introduction

Blood vessel as a closed high-pressure circulatory pathway is primarily responsible for the transport of nutrients and metabolites [1]. Blood flow homeostasis depends on a complex balance among blood cells (erythrocytes, leukocytes, and platelets), endothelial cells of the vascular lumen, blood macromolecules (plasma proteins, coagulation factors, inflammatory factors, and cytokines), and vascular mechanics [2,3]. This disruption of blood homeostasis can lead to clotting in the vascular lumen, thus hindering blood circulation [4,5]. In short, blood clots are made up of 2 main forms of blood cells, platelets and red blood cells. Thrombus formation results from a complex interaction between platelets and plasma proteins [6]. Spatiotemporal changes caused by thrombus in different parts and at different stages result in different types. Different thrombus also has different mechanisms of formation, composition, and clinical manifestation.

With the continuous deepening of thrombosis research, different types of clinical diagnosis and treatment methods are gradually being developed [7,8]. At present, the methods for clinical diagnosis of thrombosis can be divided into thrombus biomarker detection and medical imaging diagnosis [9–11]. The detection of thrombus markers mainly targets D-dimer, which can well reflect the overall activation of blood coagulation and fibrinolysis [12,13]. However, the D-dimer assay is not specific to certain thrombus types and is not accurate in detecting certain patients with special circumstances. Therefore, clinical imaging diagnosis of thrombosis has emerged. The medical diagnosis of thrombosis mainly includes computed tomography (CT) angiography [14], magnetic resonance imaging (MRI) [15], microscopic imaging [16], and color Doppler ultrasound detection [17]. These imaging methods can detect thrombosis in specific parts and have good accuracy. Although thrombus imaging has many advantages, each imaging method also has its own drawbacks. For example, color Doppler imaging is suitable for screening and surveillance but has insufficient sensitivity for stenotic venous thrombosis (VT) [18]. Angiographic imaging may cause adverse reactions such as allergy, nephrotoxicity, damage to vessel walls, and pain [19]. Clinical therapy of thrombus also includes 2 main methods: drug thrombolysis and surgical thrombectomy [20,21]. In the early stages of thrombosis suppression, anticoagulation is the main method [22]. Common anticoagulants such as plain heparin, low molecular heparin [23], and vitamin K antagonists (e.g., warfarin) [24]. Thrombolytic drugs include antiplatelet and antifibrinolytic agents such as urokinase fibrinogen activator (uPA) [25], tissue fibrinogen activator (tPA) [26], and streptokinase (SK) [27]. With the help of these medications, blood clots can be dissolved and eliminated. However, these unmodified protein drugs show greatly limited therapeutic effect due to short half-life, poor targeting ability, low accumulation efficiency, and other disadvantages. In addition, these drugs may cause unnecessary bleeding in tissues or areas and induce other serious cardiovascular diseases (CVDs) [28]. Surgical removal of the thrombus can also cause damage to the vessel wall and venous valves [29]. In short, current clinical diagnostic and therapeutic techniques still have many drawbacks in terms of targeting, permeability, stability, and biosafety. Therefore, it is important to develop new materials or new diagnostic and therapeutic methods.

With the rise of nanotechnology and nanomedicine in the last few decades, more and more micro-nano materials have emerged [30,31]. Among them, micro-nano materials can achieve effective transportation and drug delivery in the body to improve therapeutic effect. The physicochemical properties of micro-nano scale have a great impact on their interaction with the biological environment, thus determining their biological fate and drug delivery efficiency [32,33]. Improvements in the structure and properties of micro-nano materials are becoming more sophisticated in current research. Despite rapid progress in understanding the importance of the properties of micro-nano materials (e.g., shape, size, and surface charge), understanding the elasticity of micro-nano materials is not well developed. Elasticity, as an inherent mechanical property of micro-nano materials, is defined as the property of returning to the original material state upon removal of external forces [34]. Advances in materials synthesis and characterization have facilitated the study of the macroscopic role and microscopic mechanisms of elasticity, which has led to a growing interest among researchers in studying the response of material elasticity to the physiological environment. Micro-nano materials with good elastic properties can be transported and delivered in vivo through body fluids. Compared with rigid micro-nano materials, elastic micro-nano materials have good adsorption, targeting, in vivo circulation, and anti-internalization phagocytosis. These properties are consistent with the requirements of thrombosis diagnosis and drug delivery. Therefore, elastic micro-nano materials are expected to become emerging anti-thrombotic formulations.

**Fig. 1.**
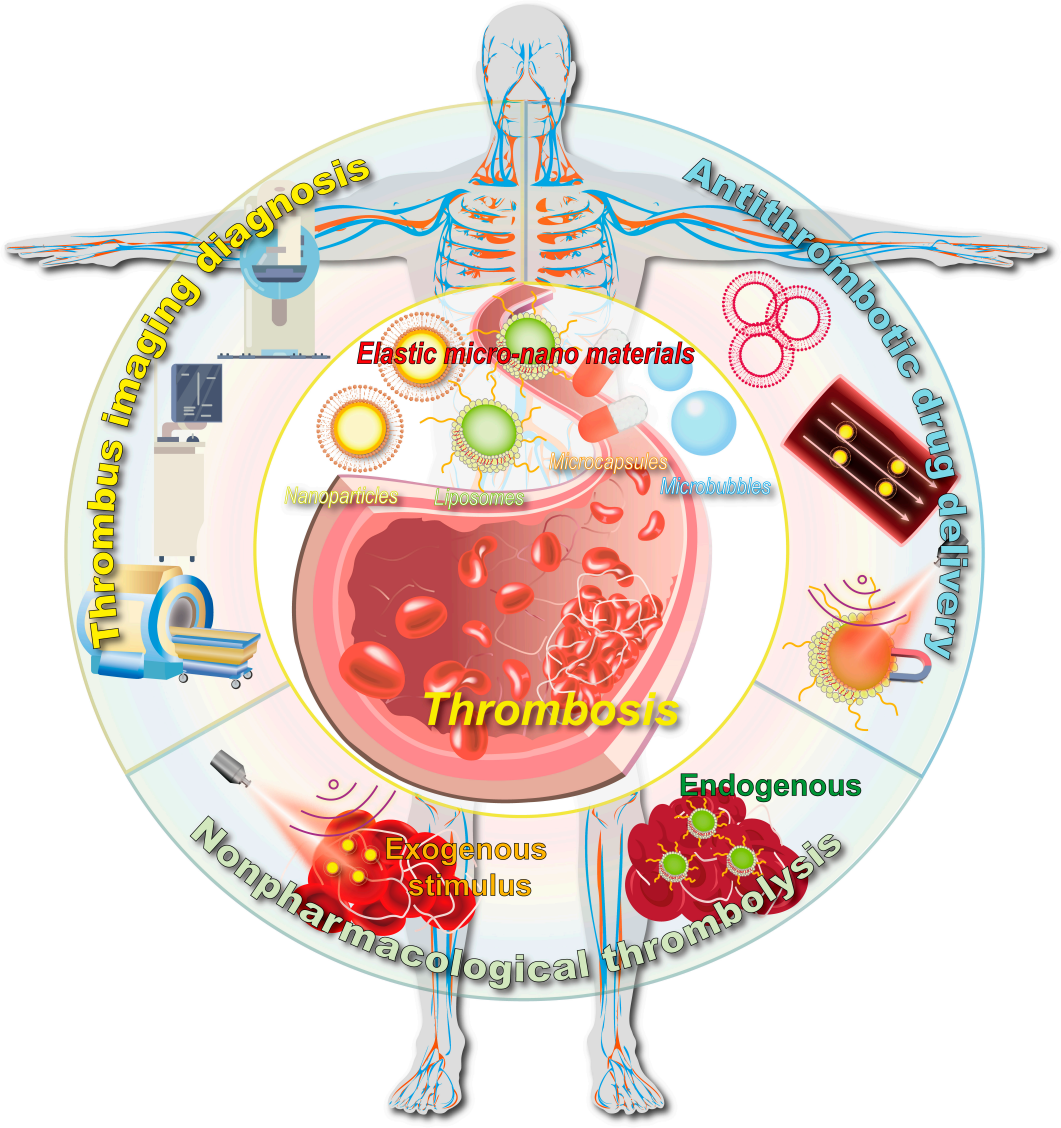
Schematic illustration of thrombosis and elastic micro-nano material thrombosis diagnosis and treatment.

Since 2011, some researchers have studied and summarized elastic nanomaterials in blood circulation, phagocytosis, endocytosis, targeting, and in vivo delivery [35–38]. Nevertheless, relevant reviews on elastic micro-nano materials in the field of thrombosis are still rare. In this review, we begin with a brief overview of thrombosis structure, formation, and possible influencing factors. Afterward, we present the basic definition of elastic micro-nano materials and synthetic strategies for regulating elasticity, and summarize elastic nano-bio interactions. As shown in Fig. 1, specialty focuses on the research and application of elastic micro-nano materials including nanoparticles (NPs), liposomes, microcapsules, and microbubbles in the thrombosis diagnosis and treatment. These materials can fine-tune the pathological occurrence and development of thrombosis to be used for thrombus imaging diagnosis, antithrombosis drug delivery, and exogenous or endogenous triggered nonpharmacological thrombolysis. This review reveals the macroscopic impact of elastic micro-nano materials in the thrombosis diagnosis and treatment and the underlying microscopic mechanisms by combining traditional and advanced diagnosis and treatment techniques. It also provides design suggestions for future elastic micro-nano materials in clinical thrombosis diagnosis and treatment. This review provides an update on the current state of research on elastic micro-nano materials, identifies emerging trends, and highlights their clinical value in the diagnosis and treatment of thrombosis in the future.

## Thrombus Microenvironment

**Fig. 2.**
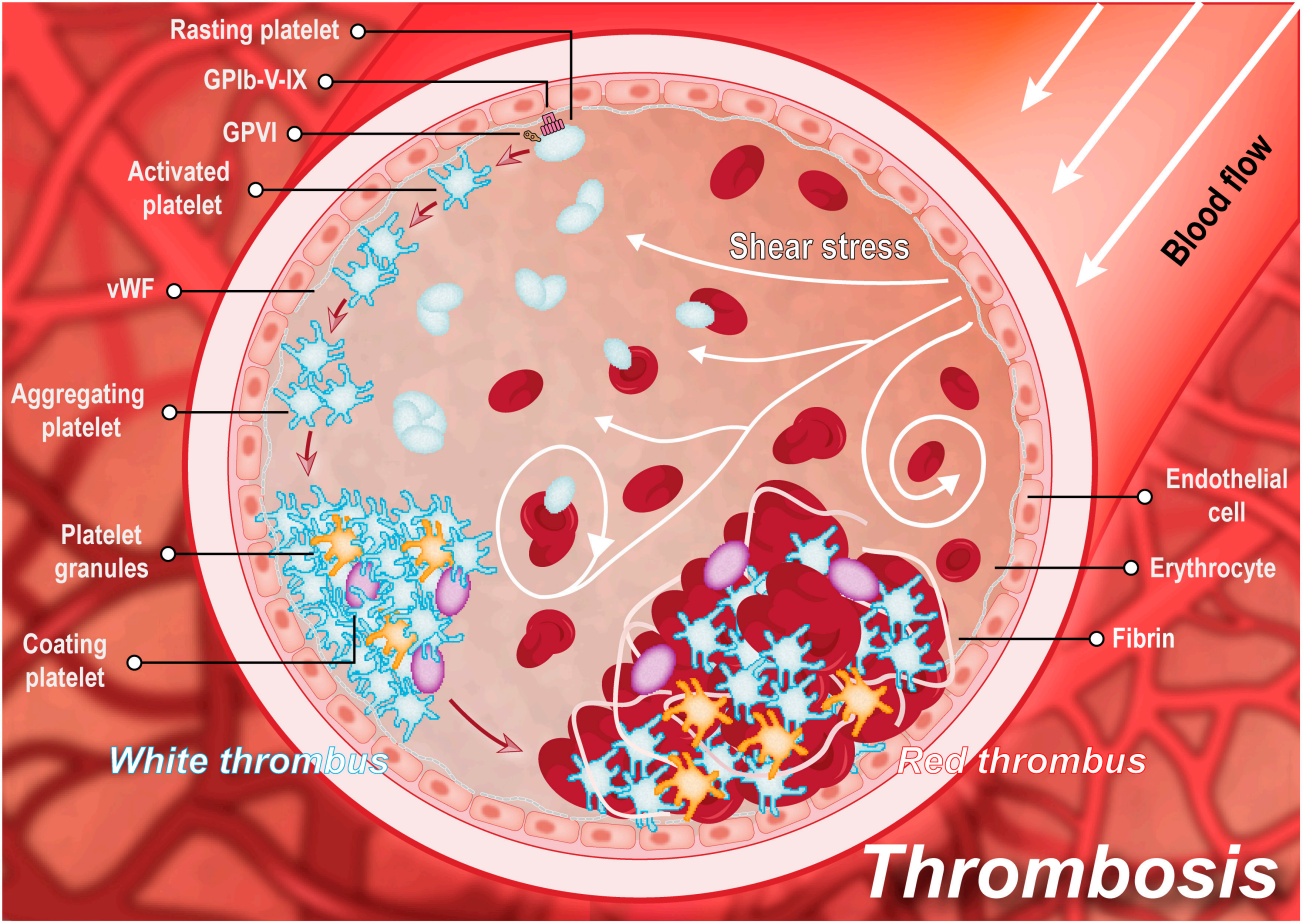
Schematic illustration of thrombosis development process and the influence of biomechanics on thrombosis.

Thrombi are small clots formed by blood flow on the surfaces of the inner surfaces of the blood vessels of the cardiovascular system at the point of denudation or repair. As shown in Fig. 2, it mainly consists of insoluble fibrin, deposited platelets, accumulated leukocytes, and trapped red blood cells [39]. The thrombus microenvironment is constructed by a combination of biomolecules, cells, tissues, and biomechanics in the thrombus environment during thrombus formation [40]. More importantly, the mechanic condition from blood flow plays an important role for the occurrence and development of thrombosis. All these complex biological and physical factors influence thrombus diagnosis and treatment process.

### Thrombosis mechanism

Thrombosis is caused by complex interactions between multiple blood components, mainly recruiting and accumulating platelets and fibrin through damaged vessel walls or activated clotting factors. Blood cells then combine with platelet surface facial mask glycoprotein (GP), plasma protein, and cross-linked fibrin to form insoluble plaque, resulting in blocking normal blood flow [41,42]. There are several main stages in the formation of blood clots.

The hemostatic system mitigates the risk of bleeding from vascular injury through the rapid response of platelets and cross-linked fibrin. However, overactivation of platelets and abnormal coagulation are the main causes of thrombus formation in blood vessels blocking blood flow [43,44]. In the early stages of thrombosis, platelets in the flow are activated by the endothelial tissue of the damaged vessel and immediately adhere to the exposed collagen fibers at the site of the injury [45]. The main proteins involved in platelet adhesion include platelet membrane GPs, von Willebrand Factor (vWF), and collagen in endothelial tissue [46]. Platelet adhesion to vascular endothelial cells depends on the interaction between multimeric vWF, which is immobilized on exposed collagen, and the adhesion platelet receptor complex GPIb–V–IX [47,48]. As an important plasma component, vWF binds to platelet membrane GP complexes and collagen fibers in vascular endothelial tissue, mediating platelet adhesion at vascular injury sites. Functional up-regulation of integrin adhesion receptors is a hallmark event in platelet activation that allows stable platelet aggregation and adhesion to endothelial cells. α_IIb_β_3_ is the most important of the integrins expressed in platelets and is primarily responsible for platelet aggregation and adhesion [49]. Upon platelet activation, integrins shift from their low-affinity to a high-affinity state, allowing integrins to bind their ligands efficiently. Ligand-occupied integrins trigger cellular processes such as cell proliferation and clot retraction [50]. Collagen-activated platelets then undergo swelling and deformation to become platelet granules while releasing active substances such as adenosine diphosphate (ADP) and promoting the synthesis of platelet-associated prostaglandin analogs [51]. ADP inhibits adenosine triphosphatase (ATPase) activity on the platelet surface and exposes it to the phospholipid surface, allowing platelets to bridge and adhere to one another. Synthetic prostaglandin analogs associated with platelets have strong platelet aggregation and vasoconstriction effects [52]. There are also other protein components involved in platelet aggregation. For example, platelet endothelial cell adhesion molecule-1 (PECAM-1/CD31) promotes cell adhesion on endothelial cells and platelets [53]. Growth arrest-specific protein 6 (Gas6) affects the secretion of platelet agonists [54]. CD40 ligand acts as a transmembrane protein on activated platelet plasma membrane that interacts with CD40 on vascular cells [55]. Protein tyrosine phosphatase-1B (PTP-1B) plays a role in platelet diffusion and clot retraction [56]. P-selectin functions as an adhesion molecule in fibronectin [57]. Platelets continuously adhere locally, forming platelet stacks, at which point platelet adhesion is reversible and can be dispersed and disappeared by blood flow [58,59]. However, with the activation of endogenous and exogenous coagulation pathways, prothrombin is converted to thrombin, which in turn converts fibrinogen to fibrin. Fibrin binds to fibronectin in the matrix at the damaged endothelium, causing an accumulation of adhesion platelets firmly anchored to the surface of the damaged endothelium, which becomes irreversible platelet thrombus and thrombus initiation point [60,61]. Afterward, the fibrin–platelet stack is released by platelet aggregation and infiltrates into a large number of red and white blood cells, forming a fibrin thrombus, which is large enough to narrow the lumen of the blood vessel and obstruct blood flow or even cause vascular embolism. Due to the obstruction of blood flow, slow flow, and blood vortex, a large number of red blood cells aggregated on the fibrin to form blood clots.

The final thrombus has different fates in the blood vessel and can be classified as softening, lysis and absorption, mechanization and recanalization, as well as calcification. Activation of fibrinolytic enzymes and leukocytes breakdown within the newly formed thrombus releases fibrinolytic proteases that soften and gradually dissolve the thrombus. The speed of thrombus dissolution depends on the size and time of thrombosis. Small and fresh thrombi are rapidly and completely dissolved; large thrombi are partially softened [62,63]. If the unabsorbed thrombus is impacted by the blood, it may form fragments or dislodge as a whole and remain with the blood flow in the vessel corresponding to the size of the thrombus, resulting in thromboembolism. The gradual replacement of thrombus by granulated tissue is called thromboembolism [64,65]. It is caused by insufficient activity of the fibrinolytic enzyme system and the prolonged presence of thrombus. After 1 to 2 d of thrombosis, granulation tissue consisting of endothelial cells, fibroblasts, and myofibroblasts grows on the vessel wall. The granulation tissue gradually penetrates deeper into the thrombus and eventually replaces it. At this point, the thrombus is firmly attached to the vessel wall and is no longer dislodged. After a certain degree of blood flow blockage, due to water absorption, thrombus drying, or partial dissolution, the surface of the thrombus will appear cracks. This phenomenon is known as thrombus mechanization [66,67]. The surrounding newborn vascular endothelial cells will be embedded and the surface of the fissure, forming new blood vessels, will be covered to restore blood flow to partially blocked vessels, a process called recanalization [68,69]. If a thrombus exists for a long time, calcium salt deposits may occur, called calcification [70,71]. After calcification, the thrombus may become a venous or arterial stone.

### Thrombotic biomechanic

Biomechanics is the science that studies the stress, deformations, and movements of living organisms, as well as their relationships with physiological and pathological environments [72–74]. Mechanical biology studies the role of mechanical factors in various life processes and the occurrence and development of related diseases throughout the organism, organs, cells, proteins, and genes. Shear stress, periodic mechanical tension, and hydrostatic pressure generated by normal blood pressure and blood flow pulsations can affect blood vessels. Among them, endothelial cells are mainly affected by shear stress, while vascular smooth muscle cells are mainly affected by periodic mechanical tension caused by pulsatile blood pressure. At the same time, various types of cells in the vascular wall are also affected by stress in the microenvironment of vascular tissue. Force-sensitive receptors on the cell surface can sense mechanical changes in hemodynamics and emit signals through adaptor molecules, thereby activating downstream signaling molecules and ultimately leading to changes in cell morphology and function.

Blood is considered as a viscous fluid that moves along the wall of a blood vessel (often considered a solid boundary) and generates shear stresses on that wall [75–77]. Shear stress is the tangential stress produced by friction of blood against the vessel wall as it flows, and is influenced by factors such as blood flow, blood viscosity, and internal diameter. Normal blood flow usually produces a shear stress of 10 dyn/cm^2^. However, thrombosis produces more complex endogenous shear stresses, which increase as the vessel diameter decreases linearly. Shear forces in the thrombotic microenvironment fall into 3 main categories: high shear stress, low shear stress, and oscillatory shear stress. High flow shear stress protects the vessel. However, low shear stress or oscillatory shear stress generated in areas of irregularly shaped vessels is not protective. Low shear stresses usually occur in the inner regions of curvature and upstream of stenoses, whereas oscillatory shear stresses usually occur at downstream of stenoses, in bifurcated sidewalls, and at branch points. Periodic mechanical tension plays an important role in cardiovascular remodeling. In the vessel wall, cyclic mechanical tension promotes vascular remodeling and contraction [78]. Hydrostatic pressure is also present in vessels with fluid [79]. Hydrostatic pressure within the normal range promotes tissue development and repair. However, pathologic hydrostatic pressures outside the normal range are capable of altering ion channel conformation and modulating ion transport across membranes, thus affecting pathophysiologic processes.

In the thrombus microenvironment, multiple thrombus components respond to shear stress to promote thrombus growth. The first step in thrombus formation is the channeling of blood components from the central stream to the periphery of the vessel wall. In the vascular system, erythrocytes in the whole blood stream have a strong influence on the movement of platelets and proteins. The flow of erythrocytes pushes the particles so that the diffusivity of the particles under shear conditions is increased. This enhanced diffusivity causes platelets to concentrate on the edge of the vessel wall [80,81]. Under the influence of high shear, vWF changes from globular to reticular, exposing platelet binding ligands that transiently bind to GPIb, the platelet adhesion receptor, ultimately leading to efficient platelet capture at the vessel wall edge [82,83]. In addition, platelet activation is also associated with high shear stress, with higher shear rates resulting in shorter platelet adsorption and activation lag times. Integrin α_IIb_β_3_ on platelets is directly modulated by high shear stress, leading to platelet activation [84]. In cases where the shear stress is sufficiently high and there are not enough α_IIb_β_3_-vWF-C1 bonds forming in all regions of the thrombus, the thrombus will fracture under high shear stress to form an embolus that occludes the vessel [85]. The appearance of an occlusive high shear thrombus at the point of occlusion is that of a white clot with almost no red blood cells. When the occlusion is complete, the surrounding blood will stagnate and a large red clot will form next to the white clot [86].

### Thrombus classification

Thrombosis can be classified as arterial thrombosis (AT) and VT depending on where they occur [87,88]. In general, AT is often accompanied by CVD. Patients with hyperlipidemia, diabetes, hypertension, chronic kidney disease, and obesity will have a marked increased risk of AT [89]. In addition, the rupture of atherosclerotic plaque can also lead to atherothrombosis. Due to the high velocity of blood flow in the arteries, even if the coagulation process is activated, enough thrombin cannot accumulate locally to form a thrombus. However, when atherosclerotic plaques collapse and endothelial cells are damaged, platelets adhere and gather, resulting in lumen stenosis and local accumulation of effective thrombin concentration, which ultimately converts fibrinogen into fibrin and forms AT [90,91]. VT usually occurs in deep veins, most commonly in the legs or arms, which is called deep vein thrombosis. Deep vein thrombosis and pulmonary embolism are collectively referred to as venous thromboembolism (VTE) [92,93]. VTE is caused by one or more factors, including slow blood flow, hypercoagulability, endothelial dysfunction, coagulation-related gene variations, endogenous anticoagulant deficiency, and nongenetic factors (immobility, surgery, and age) [94]. Thrombosis of the venous system usually occurs gradually on activated, undamaged endothelial linings, teeming with fibrin, platelets, and entrapped red blood cells.

Thrombosis is a blood clot or other blood component that accumulates together in blood vessels. Depending on the morphology, it can be classified as white, red, mixed, or transparent [95,96]. White thrombus, primarily consisting of platelets and a minimal quantity of fibrinogen, mainly forms in areas with rapid blood flow, such as heart valves, cardiac chambers, and arteries, potentially obstructing vascular lumens. Serious cases may appear diffuse intravascular coagulation or even shock [97]. Red thrombus predominantly comprises erythrocytes with a few leukocytes, all uniformly dispersed within a fibrin network. It is mainly found in veins with slow blood flow. As the water inside the thrombus is absorbed and becomes dry, inelastic, brittle, and friable, it can be dislodged to form an embolus [98]. Mixed thrombus consists of white thrombus and red thrombus and occurs in veins with slow blood flow. Blood flow passes through the thrombus and forms a vortex downstream of the thrombus, causing platelets to adhere and form the head of the venous thrombus (white thrombus). At the head of the thrombus, blood flow stagnates and coagulates, and red blood cells are gradually encapsulated in reticulated fibrin to form a red thrombus. This process is repeated alternately to form a laminated mixed thrombus [99]. Transparent thrombus mainly occurs in the microcirculation of blood vessels, which can only be seen under the microscope, so it is also known as microthrombus. It mainly contained the eosinophilic homogeneous composition of fibrin, also known as fibrin thrombus, commonly found in diffuse intravascular coagulation [100].

## Elastic Micro-Nano Materials for Thrombosis

**Fig. 3.**
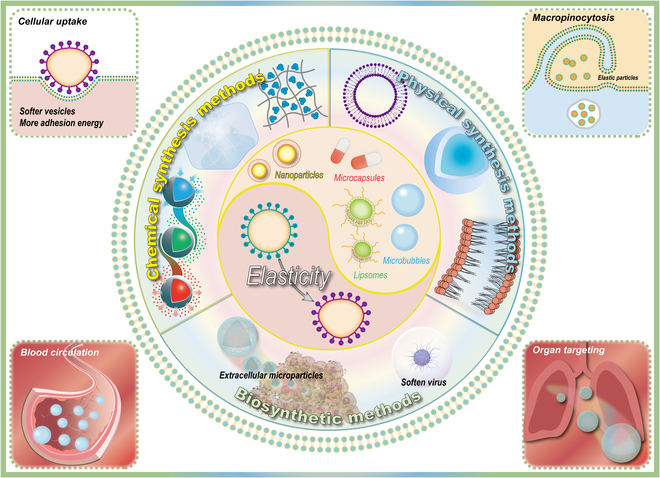
Schematic illustration of elastic modulation and in vivo fate of micro-nano materials.

The effects of NP size, shape, and surface properties on cellular uptake have been extensively studied for their basic science and translational implications. Elasticity is a key parameter in regulating NP behavior and is expected to inspire the design of more efficient nanostructures for drug delivery, biomedical imaging, and immunomodulatory therapies. For the diagnosis and treatment of thrombosis, elastic design and in vivo fate are directly related to the effect of micro-nano materials. The targeting of elastic micro-nano materials for thrombus structure and microenvironment is also a focus of research. Elastic micro-nano materials are able to undergo longer blood circulation with better targeting ability. They can be inhibited cell internalization, resulting in longer service life. As shown in Fig. 3, elastic micro-nano materials, as an emerging material, are expected to meet the needs of thrombosis diagnosis and treatment.

### Elastic micro-nano materials

Elasticity and stiffness as mechanical properties are widely used to study the deformation and softness of micro-nano materials. Elasticity is defined in physics as the property of a material to deform under the action of an external force and to regain its original size and shape when the force disappears [101]. Young’s modulus is widely used to describe the elasticity of micro-nano materials when compression is applied to them. Young’s modulus is a physical quantity of a material’s ability to resist deformation, which is described as the relationship between stress and strain during elastic deformation [102]. As the main parameter that interchangeably describes elasticity, stiffness complements flexibility or pliability and describes the property to resist elastic deformation when stressed. Unlike elasticity, stiffness is a broad structural property that is influenced by elastic modulus and geometry (e.g., size and shape) [103]. Typically, rigid or stiff micro-nano materials will have a higher Young’s modulus, while softer or elastic micro-nano materials will have a lower Young’s modulus [38].

The elasticity of micro-nano materials can be modulated by physical, chemical, and biosynthetic methods. While other physical and chemical properties (including shape, size, and surface charge) should remain unchanged. Physical methods focus on altering the structure of micro-nano materials, usually using core–shell structures to change the overall elasticity of the material [104]. Conventional physical strategies include lipid membrane coating, shell thickness modulation, and altering the amount of water between the core and shell layers [105–107]. Kong et al. [108] prepared layer-by-layer (LBL) NPs assembled from liposomal cores with layers of poly-l-arginine and hyaluronic acid. Yu et al. [109] prepared poly(lactic-co-glycolic acid) (PLGA)-lipidic core–shell NPs by a microfluidic-based system. Chemical approaches tend to microscopically alter interactions between molecules to modulate the elasticity of the material. For example, Wong et al. [110] synthesized tunable elastic poly (ethylene oxide) (PEO) hydrogel films by ultraviolet cross-linking with various concentrations of pentaerythritol tetraacrylate (PETRA) as cross-linking agent. In addition to hydrogels, Yu et al. [111] prepared liposomes with different phase transition temperatures by varying chain length and saturation of the lipid tails. Liposomes with higher phase transition temperatures have higher Young’s modulus, ranging from 2 to 27 MPa. Biological methods use elastic substances found in nature to encapsulate and modify materials. Liang et al. [112] cultured a soft tumor-repopulating cell (TRC) in soft 3-dimensional (3D) fibrin gel. TRC-derived extracellular microparticles have good elasticity and can better penetrate and accumulate in tumor tissues. In addition to biofilms, viruses are naturally occurring NPs capable of self-assembling at different stages of elasticity, providing a large resource of elastic bioparticles. Kol et al. [113] investigated the effect of elasticity regulation on human immunodeficiency virus (HIV) biological function. The HIV virus behaves stiffly during budding and softens upon entry, favoring the transmission of the HIV gene.

The study of the fate and application of elastic micro-nano materials in vivo is more challenging than in vitro studies. Micro-nano materials will interact with many organs, tissues, cells, and ubiquitous fluids in vivo. These interactions will have implications for their subsequent therapeutic modes of targeting, enrichment, or penetration. Elasticity is also a factor for influencing the propensity, kinetic rate, and internalization pathway of micro-nano materials. At the cellular level, elasticity is related to cellular uptake of micro-nano materials. For macrophages and other immune cells, harder micro-nano materials have been shown to internalize more than softer micro-nano particles. Beningo and Wang [114] prepared adjustable elastic polyacrylamide gel beads by controlling the concentration of total acrylamide to verify the mechanosensitivity of phagocytosis. The results showed a 3-fold difference in elasticity modulus between hard and soft beads. Macrophages internalized the hard beads more than the soft beads. Phagocytosis was stronger for hard beads, which was 6 times that of soft beads. For cellular uptake in membrane-encapsulated form, hard micro-nano materials require less adhesion energy than soft micro-nano materials. If the adhesion between the particles and the biofilm is not strong enough, it will be difficult for cells to swallow very soft micro-nano materials. In order to achieve complete encapsulation and endocytosis of the material, the elastic modulus of the material needs to be increased [115]. There is other literature suggesting that elasticity affects the internalization pathways of micro-nano materials at the cellular level [116]. Soft (18 kPa) NPs are internalized primarily through macropinocytosis, and stiffer NPs (35 and 136 kPa) are internalized through both macropinocytosis and clathrin-mediated endocytosis pathways, allowing stiffer nanomaterials to be ingested more efficiently by cells. In addition to cells, elasticity also affects blood circulation and organ targeting of micro-nano materials. In contrast to the efficiency of cellular internalization, soft nanomaterials have longer blood circulation times and half-lives when compared to hard nanomaterials. Anselmo et al. [37] intravenously injected soft and hard hydrogel NPs with the same radioactivity intensity but different elasticity into mice. Compared to hard NPs, soft NPs showed significantly higher persistence in the blood over a short period of time. Both their distribution half-life and elimination half-life were found to be longer. Soft NPs were retained in the circulation at higher concentrations, which may also allow for greater retention in organs with high blood flow output (kidneys, heart, lungs, and brain organs). In addition, the organ targeting ability of soft and hard NPs was verified by modifying targeting antibodies. The results showed that soft NPs significantly enhanced spleen and lung targeting compared to harder NPs. Overall, micro-nano materials with softer elasticity have longer blood circulation capacity, longer life, poorer cell internalization efficiency, and better targeting ability. These properties are useful for the use of elastic micro-nano materials in drug delivery and biomedical imaging, which can be more adaptable to thrombosis diagnosis and treatment.

### Elastic micro-nano materials for thrombosis applications

Thrombosis often causes blood vessel obstruction, stasis, and ischemic organ damage, leading to life-threatening illness or permanent disability. Given the high morbidity and mortality of thrombotic diseases, timely monitoring of thrombosis and removal of occlusive thrombosis to re-establish blood flow has been a top priority in the diagnosis and treatment of clinical thrombosis. Elastic micro-nano materials have longer blood circulation, longer life, better targeting ability, poorer cellular internalization efficiency, and better biocompatibility than conventional drugs and can be adapted to the increasingly complex needs of thrombosis diagnosis and treatment. This section summarizes recent research on elastic micro-nano materials in applications related to thrombus imaging diagnosis (in vitro imaging diagnosis, in vivo imaging diagnosis), antithrombotic drug delivery (ligand-mediated drug delivery, biomimetic drug delivery strategies, microenvironment-responsive drug delivery, thrombotic biomechanical responsive drug delivery, and exogenously stimulated drug delivery), and nonpharmacological thrombolysis (exogenously stimulated nonpharmacological thrombolysis and endogenous nonpharmacological thrombolysis).

#### Thrombus imaging diagnosis

**Fig. 4.**
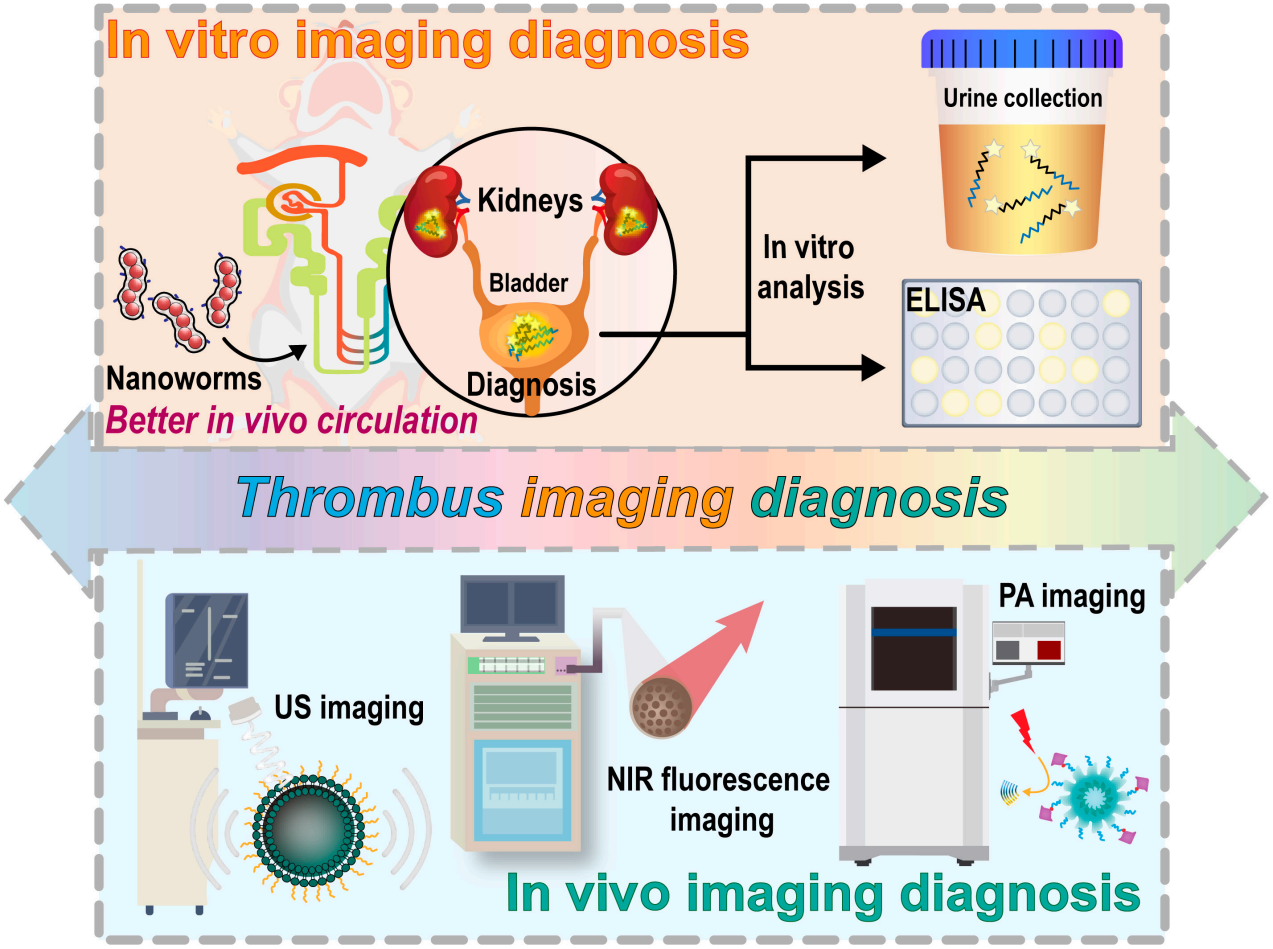
Schematic illustration of in vitro and in vivo thrombus imaging diagnosis based on different type of elastic micro-nano materials.

Thromboembolic disease is one of the current CVDs with high morbidity and mortality. Accurate detection of thrombus is a key issue in clinical practice. As shown in Fig. 4, traditional serologic and imaging methods combined with the latest molecular techniques have been widely used in clinical and research thrombus detection.

##### In vitro imaging diagnosis

**Fig. 5.**
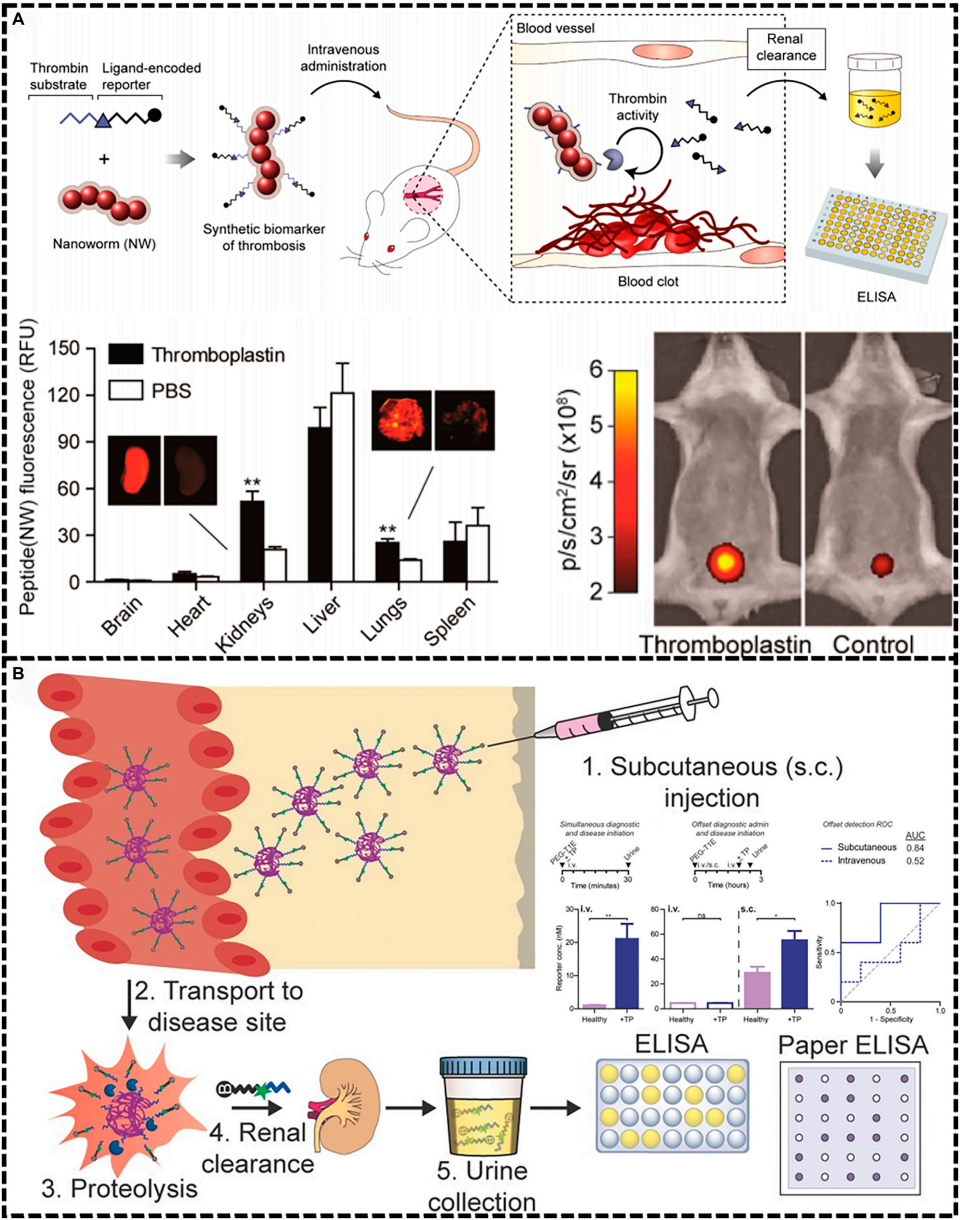
Thrombus in vitro imaging diagnosis. (A) NW detection of reporter genes in urine for thrombus imaging. Reproduced with permission from [119]. Copyright 2013, American Chemical Society. (B) Subcutaneous injection of PEG-T1E for detection of delayed pulmonary embolism. Reproduced with permission from [120]. Copyright 2016, Wiley-VCH.

With the continuous enrichment of clinical diagnosis and treatment methods, in vitro imaging as an emerging imaging method has gradually entered people’s vision. Due to its simplicity, speed, and noninvasiveness, it has been widely used in the study of various diseases [117,118]. D-dimer, an important marker of coagulation and fibrinolytic system activation, is widely used in the diagnosis of clinical thrombosis [13]. The in vitro assay is performed by collecting plasma or whole blood samples. However, the assay is still challenging due to the variability of results from hemolysis, coagulation, other diseases (rheumatoid, rubella and shingles, etc.), or interference from special populations (the elderly and pregnant women). Lin et al. [119] synthesized a nanoworm (NW) modified by a tandem peptide of a reporter gene (Fig. 5A). NW targets the site of pulmonary thrombosis with a peptide chain and releases the reporter gene into the urine in response to thrombin stimulation. The extent of pulmonary thrombosis can be verified by quantitative analysis of the reporter gene in the bladder or urine. The results show that both in vitro and in animal experiments, the amount of lung thrombus reporter genes in the presence of thrombin was significantly increased, illustrating the targeting and specificity of NW. In addition, using the elasticity of the polyethylene glycol (PEG) coating is also effective in reducing the uptake of micro-nano materials by macrophages and enhancing their ability to circulate in body fluids. Dudani et al. [120] used a PEG scaffold as a chaperone for urine biomarkers to prepare an exogenous nanosensor (PEG-T1E) (Fig. 5B). PEG-T1E also specifically responded to thrombin and was specifically detected by excreted urine. These thrombin-responsive, urine-detectable elastic micro-nano materials circulate in body fluids for long periods of time and inhibit cell internalization to some extent. However, these elastic nanomaterials with the ability to detect thrombus in vitro need to circulate in the body for a longer time. Therefore, they are unable to determine acute or early thrombosis, but can help detect thrombosis after surgery for some diseases. Different specific markers need to be established to accommodate different types of thrombi.

##### In vivo imaging diagnosis

**Fig. 6.**
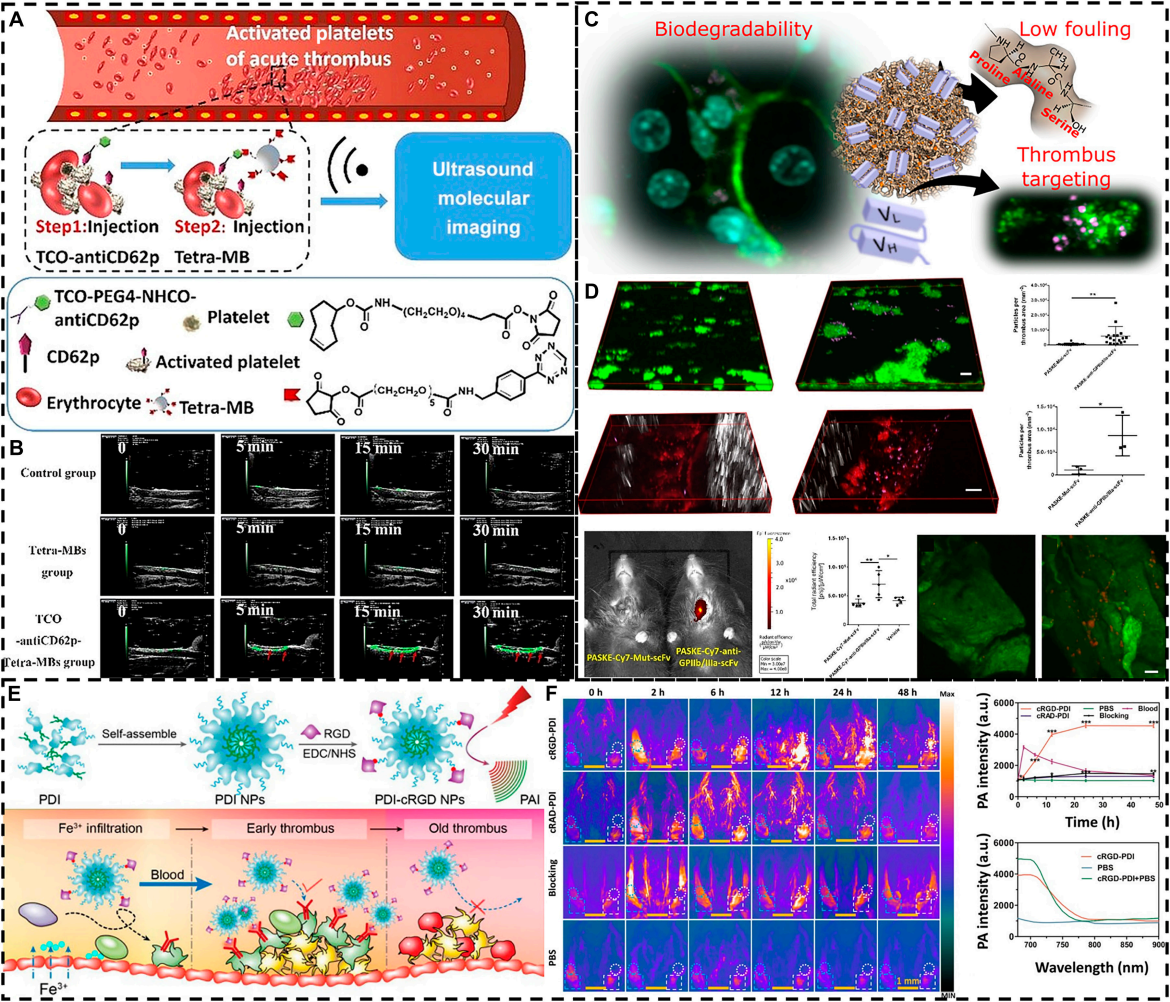
Thrombus in vivo imaging diagnosis. (A) Tetra-MBs were rapidly localized to thrombi by bioorthogonal reaction with pretargeted TCO-CD62p, and ultrasound imaging was performed for real-time imaging. (B) Ultrasound imaging of the thrombus area of ferric chloride-induced inferior vena cava (IVC) thrombosis rats during tail intravenous injection of phosphate-buffered saline (PBS) solution, with tetra-MBs without and with antiCD62p pretreatment. Reproduced with permission from [126]. Copyright 2017, Wiley-VCH. (C) Schematic illustration of low contamination and biodegradable PASKE protein particles for thrombus imaging. (D) PASKE particle thrombus imaging in a microfluidic channel thrombus model and a mouse carotid artery thrombus model. Reproduced with permission from [128]. Copyright 2018, American Chemical Society. (E) Schematic illustration of the preparation of cRGD-PDI NPs and its mechanism for specifically lightening early thrombus by PAI. (F) PAI of the mouse jugular veins with early thrombus of the cRGD-PDI NPs group, cRAD-PDI NPs group, blocking group, and PBS control group at different treatment times. Reproduced with permission from [130]. Copyright 2017, American Chemical Society.

Various imaging methods have been developed for clinical studies of thrombus imaging [121]. For example, CT imaging relies on the different densities of the thrombus and surrounding tissue, x-rays are continuously taken from different angles, and images are obtained by computer [122,123]. Angiography is performed by adding a contrast agent to a blood vessel and calculating the image by computerized sequential photography [124]. However, the conventional method has been criticized for its low sensitivity and complications caused by the contrast agent. Therefore, it is important to develop new targeting materials. Elastic micro-nano materials can effectively target thrombus sites due to their excellent blood circulation ability, which can help thrombus imaging diagnosis. Thrombus ultrasonography is currently a widely studied type of imaging. More and more researches are being done to diagnose and treat thrombus by ultrasonography through microbubbles [125]. Previously in our laboratory, a 2-step pretargeting strategy for acute thrombus molecules was used (Fig. 6A) [126]. First, TCO (1,2,4,5-tetrazine and trans-cyclooctene)-modified CD62p antibody was injected to target P-selectin on thrombus platelet granules, followed by the addition of tetrazine-labeled microbubbles (tetra-MBs) that were rapidly and selectively transported through the bloodstream to the thrombus site for ultrasound imaging. As can be seen from the results, the pretargeted group with TCO-CD62p was able to perform ultrasound imaging faster and more consistently than the group without pretreatment (Fig. 6B).

In addition to ultrasound imaging, near-infrared (NIR) fluorescence imaging provides excellent temporal and spatial resolution, fast acquisition time, ease of operation, and noninvasiveness, enabling accurate thrombus diagnosis [127]. Bonnard et al. [128] synthesized PASKE particles consisting of various amino acids (proline, alanine, serine, lysine, and polyglutamic acid) using mesoporous silica as a template (Fig. 6C). PASKE particles enable efficient molecular imaging in the practical timeframe of medical imaging. PASKE particles were then further targeted at the thrombus site by modifying the anti-GPIIb/IIIa, and Cy7 was added to further enhance the imaging capability. The results demonstrated that the final PASKE–anti-GPIIb/IIIa–scFv–Cy7 NPs formed significantly more signal in the carotid thrombus region than the nontargeted particles and carriers (Fig. 6D).

Photoacoustic imaging (PAI) with excellent spatial resolution and high optical contrast also shows more promise in thrombus diagnosis [129]. However, the lack of hemoglobin quantity and quality in the thrombus results in a diminished endogenous photoacoustic (PA) signal, which is not conducive to PAI of the thrombus region. Cui et al. [130] achieved the dual purpose of imaging and targeting the thrombus region by modifying amphiphilic stilbene 3,4,9,10-tetracarboxylic diamide (PDI) NPs with a cyclic Arg-Gly-Asp (cRGD) tripeptide (Fig. 6E). The ability of cRGD-PDI NPs to target and differentiate early thrombus from old thrombus by differentiation may be related to their strong binding capacity to GPIIb/IIIa on early thrombus-activated platelets. cRGD-PDI NPs’ PAI effect successfully provided accurate information, including contour, size, conformation, as well as spatial distribution of early thrombus, which enabled timely monitoring of the extent of thrombus obstruction and thrombolysis of blood vessels (Fig. 6F). Different types of thrombi may lead to different thrombus imaging results. Early thrombi may have more activated platelet granules. Fibrinogen immobilized on the thrombus may also bind to nonactivated platelet surface ligands, allowing late thrombi to contain more nonactivated platelets [131]. However, there is still a lack of definitions and studies on early and late thrombi. It is hoped that more diagnostic materials and tools to differentiate between early and late thrombi will be developed in the imaging field in the future in order to meet the requirements of clinical diagnosis and treatment.

#### Antithrombotic drug delivery based on elastic micro-nano materials

**Fig. 7.**
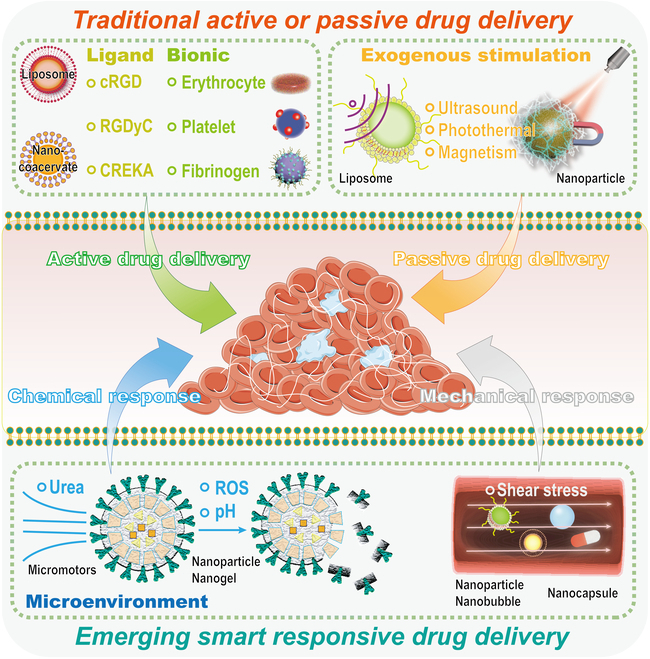
Schematic illustration of the antithrombotic drug delivery strategy based on elastic micro-nano materials.

Thrombosis treatment is most commonly carried out by drugs for anticoagulation and thrombolysis. However, due to the shortcomings of conventional drugs such as poor targeting ability, low accumulation efficiency, and side effects, the final thrombosis treatment effect will be affected. Therefore, it is of great significance to construct new and effective drug delivery systems. Traditional active drug delivery includes ligand-mediated drug delivery and bionic drug delivery. Such drug delivery strategies involve combining drugs with targeted macromolecules (antibodies and peptides) for precision therapy. Traditional exogenous stimulus-responsive drug delivery systems (light, magnetic, and ultrasound) are also advancing in thrombosis diagnosis and treatment. With the increasing research on the vascular and thrombus microenvironment, microenvironment-responsive drug delivery systems, especially with biomechanically responsive elastic micro-nano material drug delivery systems, are emerging. The currently studied elastic micro-nano materials are widely investigated in the field of drug delivery, which can be well combined with antithrombotic drugs and play a role. As shown in Fig. 7, we will next focus on traditional and emerging drug delivery strategies for elastic micro-nano materials. A comprehensive introduction to elastic micro-nano materials for thrombotic drug delivery applications through traditional active drug delivery, traditional passive drug delivery, and emerging smart responsive drug delivery strategies has been summarized and envisioned.

##### Traditional active drug delivery

**Fig. 8.**
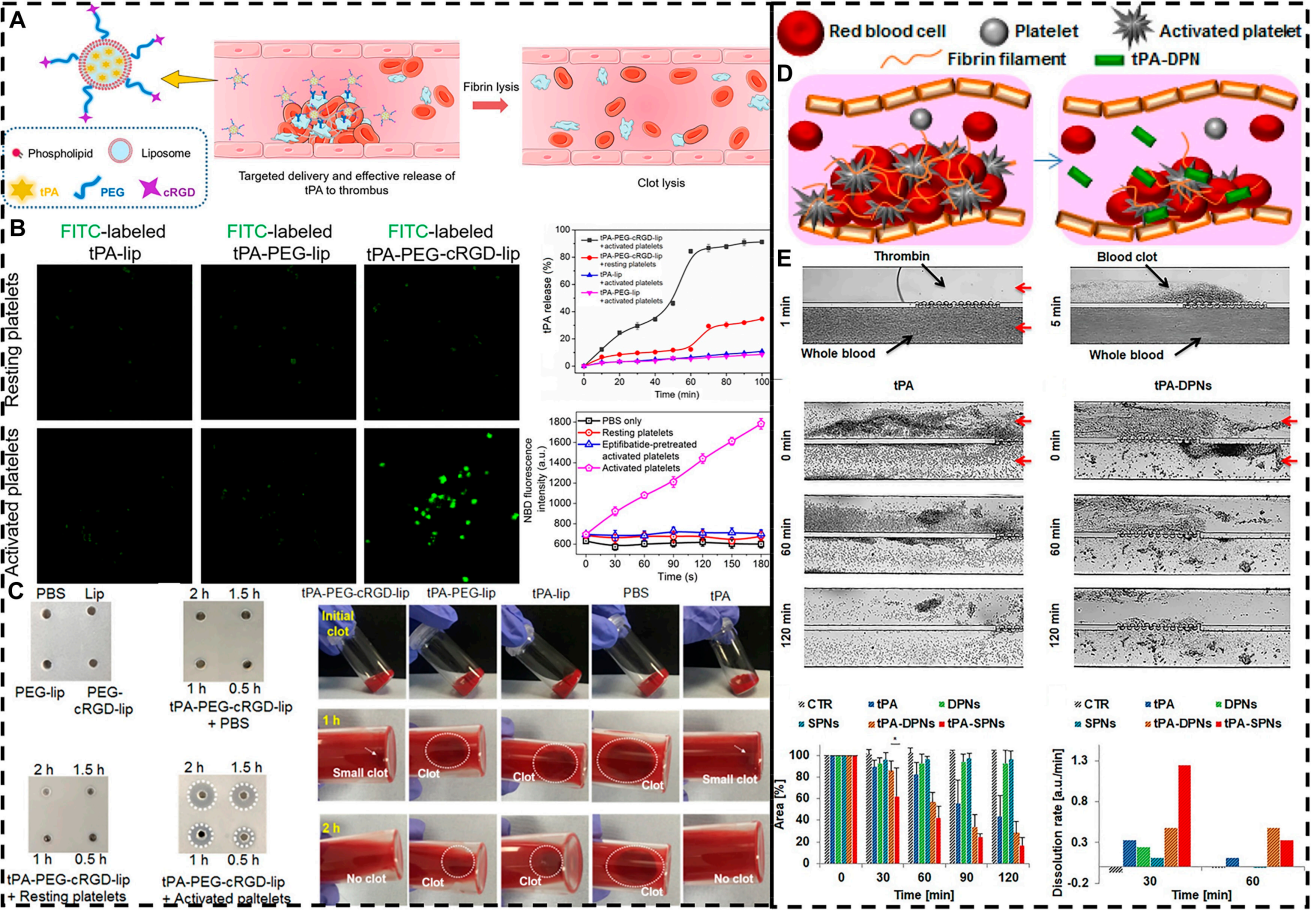
Traditional active drug delivery for thrombolysis. (A) Schematic illustration of targeted delivery and release of tPA specifically at the thrombus site using tPA-PEG-cRGD-Lip. (B) tPA-PEG-cRGD-Lip activates platelet targeting, drug release, and membrane fusion ability. (C) tPA-PEG-cRGD-Lip drug thrombolysis. Reproduced with permission from [133]. Copyright 2019, Elsevier B.V. (D) Schematic illustration of tPA-DPN-targeted drug thrombolysis. (E) In vitro blood clot elimination efficacy of tPA-DPNs and tPA-SPNs under dynamic conditions. Reproduced with permission from [139]. Copyright 2018, American Chemical Society.

As time goes on, traditional thrombotic drug delivery systems need to become more precise and personalized. Drugs need to be tailored to each patient’s needs and then delivered specifically to the disease lesion site. Achieving this precise targeting strategy requires linking the drug to a specific ligand that can selectively attach or react in the focal area [132]. Elastic micro-nano materials such as liposomes are able to encapsulate drugs well and have a good affinity for the targeted ligand. Huang et al. [133] developed a multifunctional liposome system that is capable of selective targeting and effective thrombolysis while minimizing adverse side effects (Fig. 8A). Hydrated lipid membranes were prepared by mixing l-α-phosphatidylcholine (EPC), cholesterol, and 1,2-distearoyl-sn-glycero-3-phosphorylethanolamine (DSPE)-PEG-NH_2_-modified cRGD in a chloroform–ethanol binary organic system. Finally, tPA was encapsulated to synthesize tPA-PEG-cRGD-Lip (liposome). tPA-PEG-cRGD-Lip was able to target activated platelets at the thrombus site very well and release tPA efficiently through membrane affinity and membrane fusion (Fig. 8B). Compared with unmodified cRGD and free tPA, tPA-PEG-cRGD-Lip was able to effectively release tPA through membrane affinity and membrane fusion, and possessed good thrombolytic activity, which could significantly shorten the thrombolytic time (Fig. 8C). All these results demonstrated that the use of liposomes to encapsulate the drug and modify the targeting ligand can significantly improve the thrombolytic effect and reduce thrombolysis complications, such as bleeding. In addition to liposomes, polymeric micro-nano materials directly composed of drug-targeting ligands are more resistant to mechanical forces and have better drug activity than liposomes. Huang et al. [134] synthesized RGD-Chi@tPA by combining positively charged chitosan-modified RGD (RGD-Chi) and negatively charged tPA via electrostatic interaction forces. Compared to free tPA, RGD-Chi@tPA significantly prolonged circulation time and demonstrated effective thrombus targeting and penetration, enabling complete revascularization. These polymeric delivery platforms, constructed by electrostatic interaction forces, are able to release drugs more efficiently and retain drug activity under the blood flow condition. Elastic micro-nano materials can modify ligands to have good thrombus-targeting ability and increase blood clearance half-life and drug retention by binding or wrapping the drug, thus improving the thrombolytic effect.

Bionic strategies are inspired by nature and can mimic biomaterials not only by their chemical structure but also by their biological function. Biomimetic strategies not only have good biocompatibility and biodegradability that can degrade the risk of immunogenicity of the drug in the body but also can prolong the circulation time of the drug in the body [135,136]. The most common biomimetic strategy for CVDs such as thrombosis mimics the circulation of erythrocytes in the body to target lesions [137,138]. Colasuonno et al. [139] prepared soft disk-like polymer nanostructures (DPNs) with a diameter of 1,000 nm and a height of 400 nm by a top-down lithographic synthesis strategy using erythrocytes as templates (Fig. 8D). DPN was then directly coupled with tPA for drug loading (tPA-DPN). The porous matrix of tPA-DPN allows better tPA loading and increases the circulating time of tPA in vivo. In addition, positively charged tPA-DPN can adsorb negatively charged components of the circulating thrombus to target the thrombus. tPA-DPN has a weaker thrombolytic capacity in the microfluidic system than spherical polymeric nanoconstructs tPA-SPN. However, tPA-DPN has a better thrombolytic capacity in the animal thrombus model than tPA-SPN in disk form (Fig. 8E). Differences in thrombolytic effects illustrate the role of bionic structures in humoral circulation. In addition to erythrocytes, activated platelets are also a major thrombi component [140,141]. Chen et al. [142] constructed a phototherapeutic thrombolysis platform by hybrid self-assembly of cotton ball-shaped platelets (PLTs), P6 (hirudin P6), and PEDOT [phototherapeutic poly (3,4-ethylenedioxythiophene)] to form the P6@PEDOT@PLT nanomotor (NM). P6@PEDOT@PLT achieves thrombus targeting by adhesion of P-selectin on platelet membranes. Xu et al. [143] prepared an engineered nanoplatelet (PNP-PA) that can be drug-loaded by surface coupling to tPA. PNP-PA exhibited effective innate targeting and local clot degradation and was able to reduce the risk of hemorrhage by evading the systemic fibrinolytic state. In addition to platelets, targeting by affinity between fibrinogen and thrombus-activated platelets is also a viable strategy [144,145]. Ye et al. [146] prepared microcapsules by immobilizing fibrinogen on the surface of a polydopamine (PDA) layer of polystyrene microspheres (PS), which would subsequently be loaded with natriuretic kinase (NK) by diffusion. PDA-Fib-NK microcapsules have good targeting of thrombus-activated platelets. In terms of antithrombotic activity, microcapsules also have strong fibrinolytic properties and low side effects. Biomimetic strategies can further improve the blood circulation time of elastic micro-nano materials and enhance the biocompatibility and targeting ability of the materials.

The specificity of thrombus targeting and the feasibility of drug release remain concerns for traditional active drug delivery systems. Targeting ligands and biomimetic strategies for different thrombus sites and periods need to be further subdivided. It is hoped that more specific thrombus drug delivery systems can be developed in the future.

##### Traditional passive drug delivery

Due to the dense fibrin network on the thrombus surface and the surrounding shear forces, existing thrombotic drugs, including nano-delivery particles, often remain on the thrombus surface and are difficult to penetrate [147,148]. When a drug cannot penetrate a thrombus through its structural targeting, delivery of the drug can be achieved with the help of exogenous stimuli [149,150]. External stimuli (e.g., light, magnetic, or ultrasound) allow controlled drug release from the nanocarrier with spatiotemporal resolution [151]. Traditional passive drug delivery overcomes the problems associated with nanodrug specificity. The main drug delivery NMs that have been extensively studied include magnetic-driven, ultrasonic (US)-driven, and photothermal-driven.

**Fig. 9.**
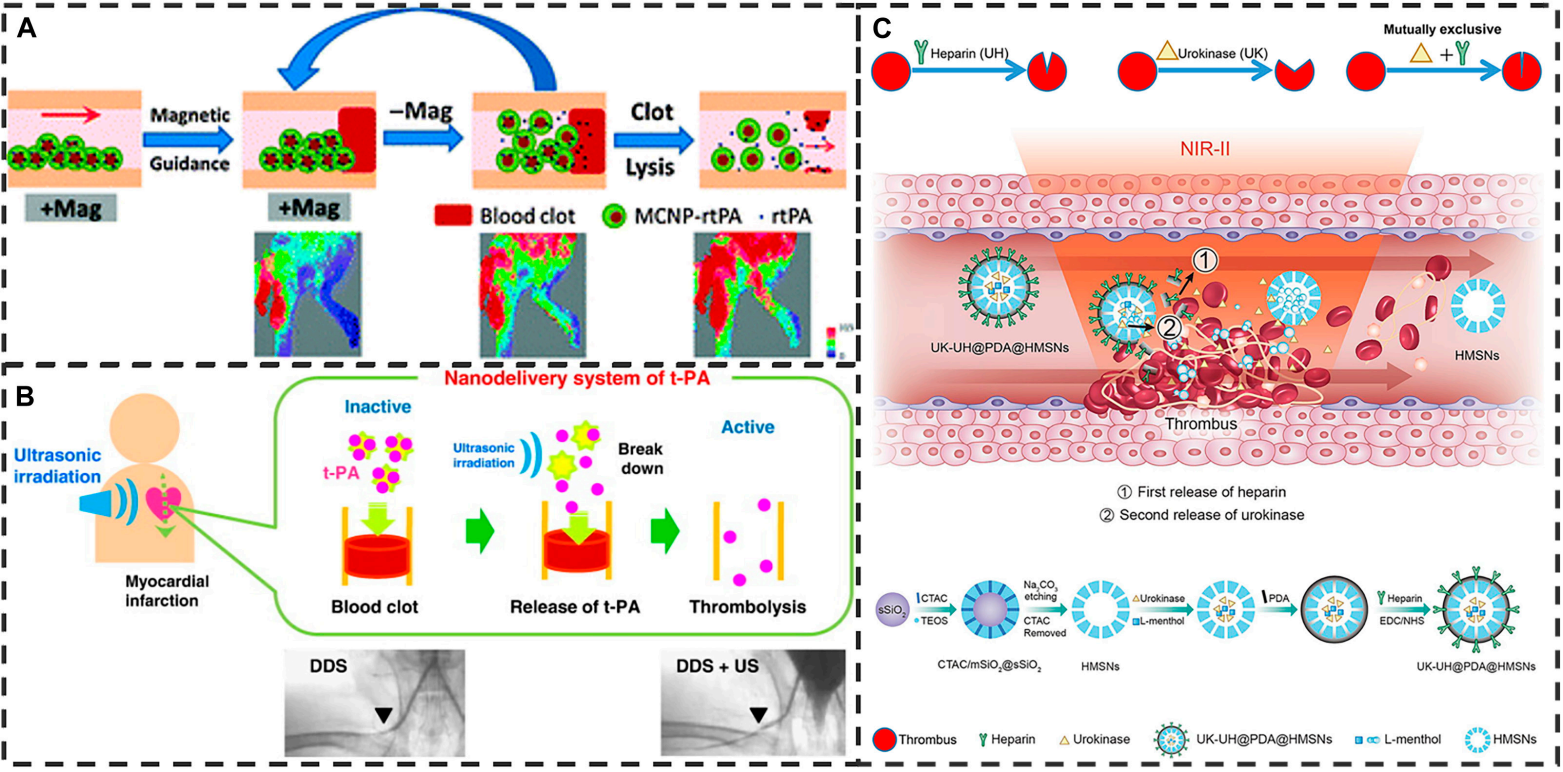
Traditional passive drug delivery for thrombolysis. (A) Schematic diagram of MCNPs-rtPA magnetically guided drug release thrombolysis. Reproduced with permission from [154]. Copyright 2016, The Royal Society of Chemistry. (B) Schematic diagram of the ultrasound-induced release of rtPA thrombolysis by the nanodelivery system. Reproduced with permission from [157]. Copyright 2010, Elsevier B.V. (C) Schematic diagram of photothermal-induced dual-drug ordered release thrombolysis of UK-UH@PDA@HMSNs nanosystem. Reproduced with permission from [162]. Copyright 2020, American Chemical Society.

Magnetic actuation is a conventional actuation method in which drug micro-nano materials are actuated to target thrombi in the presence of an external magnetic field [152,153]. Chen et al. [154] prepared magnetic chitosan NPs encapsulating rtPA (MCNPs-rtPA) by ion gelatinizing water-soluble chitosan (WSC) with tripolyphosphate (TPP) in the presence of rtPA and Fe_3_O_4_ magnetic NPs to achieve controlled release of rtPA (Fig. 9A). MCNPs-rtPA, due to its magnetic-sensitive release property, can be guided to the thrombus site and can release rtPA in response to a moving magnetic field to achieve thrombolysis.

Ultrasound, as a common therapeutic tool, is capable of thrombolysis through acoustic flow mechanisms, radiation, and US cavitation. However, its application alone is limited by the problems of embolization of thrombus fragments and vascular injury that may result from mechanical forces [155,156]. In contrast, motion targeting and drug release by US-powered elastic micro-nano materials have been increasingly studied. The combination of US and micro-nano drug platforms is one of the current trends. Uesugi et al. [157] designed a tPA nanoscale delivery system with thrombolytic activity that inhibited tPA with no ultrasound treatment, while its activity was restored only after exposure to ultrasound (Fig. 9B). In a rabbit model of thrombosis, intraventricular administration of the PEG-modified complex followed by ultrasound irradiation resulted in complete recanalization, in contrast to administration of the complex alone. Also, Tiukinhoy-Laing et al. [158] reported an echogenic liposome loaded with tPA, which ruptured under ultrasound to release tPA, leading to enhanced clot thrombolysis.

In addition to magnetism and ultrasound, photothermal as an exogenous stimulus with strong penetration has also been widely used in antithrombotic NMs [159,160]. Cao et al. [161] developed a PLGA NP (GPRPP-Y8U@P). GPRPP-Y8U@P was surface modified with the fibronectin-targeting peptide Gly-Pro-Arg-Pro-Pro (GPRPP), centered with the phototherapy diagnostic agent Y8 and uridine kinase (UK). GPRPP-Y8U@P shows significantly enhanced pharmacological thrombolytic ability after NIR irradiation. Zhong et al. [162] constructed a multi-functional dual-drug sequential thrombolytic release platform (UK-UH@PDA@HMSNs) (Fig. 9C). UK-UH@PDA@HMSNs consisted of PDA-modified hollow mesoporous silica (HMSN) loaded with UK and ordinary heparin (UH) with dual physical assistance (NIR-II and bubbles). With the help of a NIR-II (1,064 nm, 1.0 W cm^−2^) laser, the photothermal effect of the PDA can be stimulated to promote the UH release, thus accelerating thrombolysis. Subsequently, the localized thermal effect can accelerate the phase transition of l-menthol in HMSN, which generates bubbles to promote the release of UK, thereby achieving the sequential release of the 2 thrombolytic drugs.

Except for single exogenous stimulation therapy, the multimodal fusion of different exogenous triggers is currently the focus of pharmacological thrombolysis research. Ruan et al. [163] designed a novel NM by combining iron oxide/perfluorohexane (PFH)/UK into liposomal nanovesicles. NM was transformed by NIR/US induction and could be used for noninvasive intravenous thrombolysis. NM was effectively aggregated at the thrombus site under magnetic field guidance. Subsequently, NIR/US stimulation drove PFH to produce a phase transition and generate a cavitation effect to induce NM penetration deeper into the thrombus. Ultimately, UK is released from the collapsed NM and drug thrombolysis is achieved synergistically.

Traditional passive drug delivery systems are capable of more effective targeted penetration of nanomedicines free from the constraints imposed by the thrombus microenvironment. However, the penetration and effectiveness of exogenous stimulation need to be verified at a deeper level, and side effects such as heat and radiation need to be further verified for their safety.

##### Emerging smart responsive drug delivery

Current conventional drug delivery systems can theoretically deliver all drugs. However, their clinical performance needs to be further evaluated due to the possible adverse effects of ligands and the penetration depth of exogenous stimuli. In addition, mediocre micro-nano carriers are often not as effective as they should be in complex internal environments. Smart nanoplatforms for microenvironmental response are in urgent need [164]. In recent years, smart drug release strategies based on the disease microenvironment have been extensively studied [165,166]. Smart drug delivery systems can be categorized into chemically responsive and mechanically induced microenvironmental drug delivery. Disease microenvironment-responsive drug delivery systems can reduce problems such as bleeding caused by off-target drugs or drug inactivation due to short half-lives [167,168]. Therefore, it is of great importance to construct selective release systems according to different thrombotic microenvironments.

**Fig. 10.**
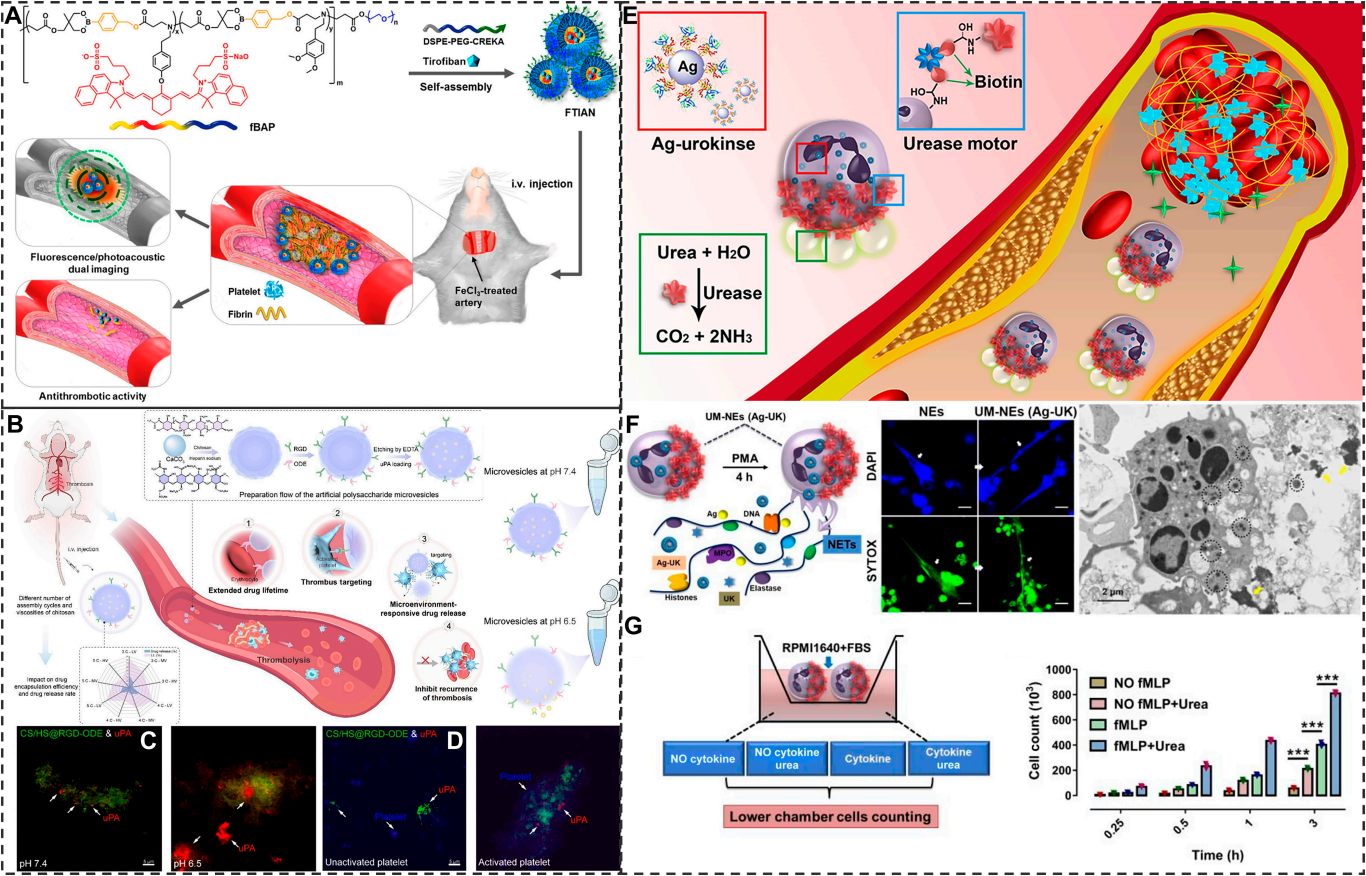
Chemically responsive smart drug delivery for thrombolysis. (A) Schematic diagram of FTIAN as a thrombus-specific theragnostic agent. Reproduced with permission from [171]. Copyright 2017, American Chemical Society. (B) Schematic illustration of uPA-CS/HS@RGD-ODE for prolonged circulation time, targeted drug delivery, microenvironmental responsive drug release, and inhibition of thrombus recurrence. (C) pH-responsive uPA release. (D) Platelet tension-responsive uPA release. Reproduced with permission from [176]. Copyright 2024 Elsevier Ltd. (E) Schematic design of urease motor-driven NE drug delivery system UM-NEs (Ag-UK) for drug thrombolysis. (F) Drug release from the UM-NEs (Ag-UK) system. (G) In vitro inflammation and urea-driven targeting capability of the UM-NEs (Ag-UK) system. Reproduced with permission from [179]. Copyright 2022, American Chemical Society.

Chemically responsive smart drug delivery can be divided into reactive oxygen species (ROS)-responsive, pH-responsive, and urea-driven elastic micro-nano material systems. During thrombosis, ROS play an important role in platelet activation and aggregation [168,169]. ROS are produced in thrombi by injured endothelium and activated platelets. Elevated ROS levels in turn further lead to endothelial dysfunction and platelet activation, which promotes thrombus propagation. ROS mediates endothelial expression of inflammatory cytokines and promotes platelet–endothelial interactions and vascular occlusion. Therefore, controlling or eliminating ROS in thrombus is also one of the elements to be taken care of in thrombus therapy. In addition, elevated ROS levels in the thrombus microenvironment can be used as a switch for controlled drug release. Zhao et al. [170] developed a dextran–tirofiban-coupled NP (T-RBC-DTC NP), which was further encapsulated by erythrocyte membranes and modified with CREKA peptide. T-RBC-DTC NP was able to target the thrombus site very well and reacted with a high concentration of H_2_O_2_ at the thrombus site to release the antiplatelet drug tirofiban. Erythrocyte membranes and modified CREKA peptides are thrombus-targeting components, and dextran is an H_2_O_2_ reactant. T-RBC-DTC NP had good H_2_O_2_ scavenging ability and was able to target the thrombus site better than RBC-DTC NP. In the thrombus model, T-RBC-DTC NP demonstrated excellent thrombolytic ability and almost complete revascularization. Kang et al. [171] developed a fibrin targeting and antithrombotic nanomedicine (FTIAN) (Fig. 10A). FTIAN is capable of thrombolysis by targeting fibrin via CREKA peptides and releasing the antiplatelet drug hydroxybenzyl alcohol (HBA) through the reaction of boronate antioxidant polymers (fBAP) with H_2_O_2_. FTIAN has a favorable thrombolytic effect in animal models. In addition to ROS, the pH of the thrombus site is another factor to be considered. Due to oxygen depletion and preferential metabolism by anaerobic glycolysis, pH in ischemic tissues decreases and the thrombotic microenvironment becomes weakly acidic [172,173]. Cui et al. [174] prepared a PEG-coupled urokinase nanogel (PEG-UK) responsive to pH gradient differences. One hour after the middle cerebral artery occlusion, the pH of the occluded area decreased from normal 7.22 to 6.73. The weak acidic environment allowed the PEG-UK hydrogel to rapidly release the thrombolytic drug, and the volume of cerebral arterial embrasure gradually decreased with relatively low neurological deficits. Li et al. [175] coupled a fluorescein isothiocyanate-labeled uPA to oxidize dextran (Oxd) via pH-sensitive imine binding and then modified the coupler with an RGD peptide to synthesize nanomaterials (FITC-uPA-Oxd-RGD) with a targeted and weak acid-responsive phenotype. FITC-uPA-Oxd-RGD reaches normal pH because the entangled polymer layer is not subjected to hydrolysis by proteases. In the weakly acidic thrombus environment, FITC-uPA-Oxd-RGD hydrolyzes to release uPA for the thrombolytic effect. Shan et al. [176] constructed an artificial polysaccharide microbubble drug delivery system (uPA-CS/HS@RGD-ODE) (Fig. 10B). uPA-CS/HS@RGD-ODE prolongs circulation time by coupling 2-(N-oxidized-N, N-diethylamino) ethyl methacrylate (ODE) to erythrocytes, targets thrombus via RGD, and releases uPA via the chitosan–H_2_O_2_ reaction at the thrombus site (Fig. 10C and D). Urea is widely available in the blood as a small-molecule metabolic end product and is a biofuel studied by many self-propelled micromotors [177,178]. Zheng et al. [179] immobilized urease asymmetrically on the surface of natural neutrophils (NEs) and then loaded UK-coupled silver (Ag) NPs (Ag-UK) to obtain an urease catalysis micromotor-powered (UM)-NEs (Ag-UK) system driven by self-propelled micromotors (Fig. 10E). NEs can actively target thrombi and release NE extracellular trap reticulum (NET) to promote thrombus expansion (Fig. 10F). Thus, NEs can be excellent carriers of thrombolytic drugs. In addition, urease catalyzes the production of ammonia and carbon dioxide from endogenous urea to generate thrust, which propels NEs to rapidly target thrombi (Fig. 10G). UM-NEs (Ag-UK) showed significantly faster thrombolytic capacity compared to the other 3 groups of NEs (Ag-UK), Ag-UK, and UK.

**Fig. 11.**
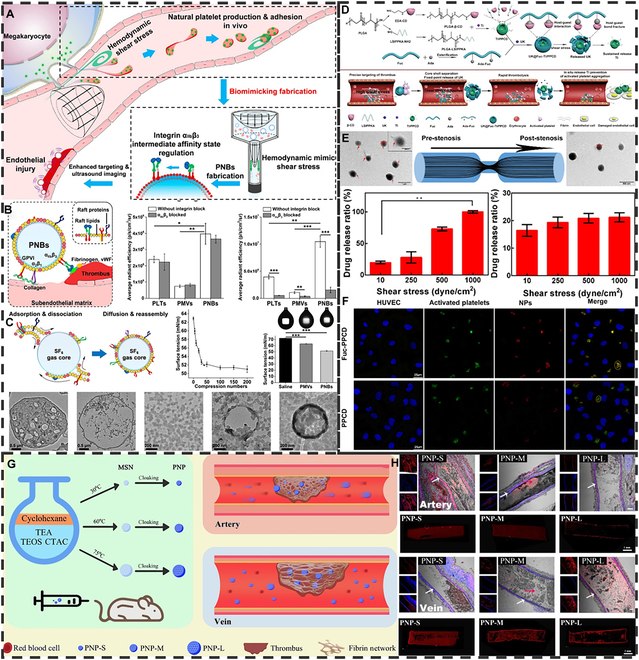
Mechanically induced smart drug delivery for thrombolysis. (A) Schematic diagram of the relationship between platelets and biomechanical signals and the fabrication and function of PNBs. (B) Specific adhesion of PNBs to subendothelial matrix and thrombus. (C) The assembly process of PMVs’ fragments on the surface of nanobubbles. Reproduced with permission from [185]. Copyright 2022, American Chemical Society. (D) Schematic illustration of the synthesis route, drug release mechanism, and targeted thrombolysis of UK@Fuc-TI/PPCD. (E) UK@Fuc-TI/PPCD drug release in response to shear stress. (F) The colocalization ability of Fuc-PPCD NPs with activated platelets. Reproduced with permission from [186]. Copyright 2021, Elsevier Ltd. (G) Schematic illustration of the penetration and retention of the platelet membrane cloaking NP with different sizes in arterial and venous thrombus. (H) NP enrichment in static blood clots, arterial thrombus, and venous thrombus. Reproduced with permission from [189]. Copyright 2022, Elsevier B.V.

Mechanically induced smart drug delivery systems using elastic micro-nano materials primarily operate through the high shear stress generated by blood at the thrombus site. This shear stress either activates target ligands or directly enhances material permeation and accumulation. Thrombosis leads to a reduction in the vessel’s cross-sectional area, which leads to an increase in site-specific shear stress, and pathological shear further promotes thrombosis and vascular remodeling [180]. High shear also affects platelet activation and aggregation. Platelets can quickly sense and respond to hemodynamics, allowing the mechanical environment to regulate their activation [181]. When vascular stenosis occurs, an increase in shear stress caused by a local disturbance in blood flow results in mechanical sensing of integrin α_IIb_β_3_ on platelets as well as activation of the corresponding ligands (e.g., fibrinogen, fibronectin, and vascular hemophilic factor) [182,183]. The use of such high shear stress for targeted and controlled drug release therapy has become a focus of current research. Molloy et al. [184] prepared a shear stress-sensitive phosphatidylcholine (PC)-based nanocapsule. The nanocapsule was able to release the antiplatelet drug integrin in response to pathological shear stress. The nanocapsule can release integrin to prevent platelet aggregation for high shear-induced thrombolysis in both a microfluidic model and a robust AT model. Li et al. [185] designed a platelet mimetic nanobubble (PNB) with a dual targeting function, which is expected to respond to high shear stresses and be able to adsorb thrombi through the platelet membrane (Fig. 11A). In a tunable and repetitive pressure system device consisting of a syringe and a vial, high shear stress resulted in the formation of nanoscale SF_6_ (sulfur hexafluoride) free bubbles and fragmentation of platelet membrane vesicles (PMVs). Then, driven by surface tension and hydrophobic forces, PMV fragments were adsorbed on the surface of SF_6_ nanobubbles formed by stabilized PNBs. Under repetitive pressure changes, the conformation of integrin α_IIb_β_3_ on the surface of PNBs shifted to an intermediate affinity state, which in turn enhanced the targeting adhesion ability of PNBs (Fig. 11B and C). Zhang et al. [186] constructed an “on-off” drug library capable of accurately recognizing thrombi and responding to changes in shear stress (Fig. 11D). Core–shell NPs based on oligofructose sulfate (Fuc) and poly lactic-co-glycolic acid (PLGA) core (PPCD) were prepared by β-cyclodextrin (β-CD) host–guest inclusion interactions. The thrombolytic drug UK and the antiplatelet drug tirofiban (TI) were loaded into the shell and core, respectively. Once at the thrombus site, UK@Fuc-TI/PPCD can be precisely targeted by recognizing the P-selectin of activated platelets in the thrombus region. Then, the sharply increased shear force at the targeted thrombus breaks the core–shell structure to rapidly release UK for site-specific thrombolysis. Subsequently, TI contained in the PPCD core is slowly released at the thrombolytic site to prevent re-embolization of the vessel (Fig. 11E and F). Griffin et al. [187] evaluated the effect of carboxyl-modified charged NPs (CNPs) on AT in high shear flow. In the microfluidic model, the physical interaction of charged CNP with vWF or platelets under shear flow decreases arterial thrombus. In addition to the chemical response to platelets and elastic micro-nano materials through thrombus shear, physical effects are also one of the factors to be considered for target materials. Different types of thrombi will also have an impact on the targeting effect of materials [188]. Wang et al. [189] verified the permeation differences in arterial thrombus and venous thrombus by preparing platelet-encapsulated mesoporous silica NPs with different sizes and dimensions (Fig. 11G). The results showed that in the static clot state, the larger the particle size of the NPs, the poorer their permeability and the smaller the degree of accumulation in the clot. However, the results were different due to the effect of blood shear. In venous thrombi, the larger the particle size of NPs, the more NPs accumulate. The larger pore size of the clot in venous red thrombus will force the larger NPs to penetrate the clot under higher thrombus shear, while the smaller NPs will be washed away. However, the effect is reversed in arterial white blood clots. White blood clots have smaller pore sizes, and more small particles will be pushed through by high shear stresses, while large particles will be washed away directly from the edges (Fig. 11H). Based on the different penetration and clearance abilities of different particles under shear stress, elastic micro-nano particles with targeted structures can be prepared for targeting and penetration according to the type of thrombus.

The smart response drug delivery strategy of elastic micro-nano materials not only prolongs the blood half-life of drugs and reduces internalized clearance by endothelial cells but also selectively responds to the complex and dynamic thrombotic microenvironment network for chemical or mechanical drug release. However, the microenvironment may change accordingly during different thrombotic periods, and the emergence of oxidative stress in the thrombus region may also cause inflammation. Therefore, the prepared elastic micro-nano materials should also have a certain anti-inflammatory ability. The pH of the thrombus region may remain at normal levels in the early stages, and the thrombus occludes the blood vessel in the late stages before it becomes weakly acidic. In addition, the complex mechanical environment surrounding the thrombus changes over time. Clinical translation of the shear stress response of elastic micro-nano materials is still limited by the lack of suitable in vitro and in vivo models to simulate the human blood microenvironment. Current microfluidic systems lack the influence of vascular endothelial cells on elastic micro-nano materials, while mouse models in vivo do not fully simulate the human blood microenvironment. In summary, the design of smart microenvironment-responsive elastic micro-nano materials still needs to take into account the variation of multiple factors. The design of multifactorial responsive elastic micro-nano material drug delivery platforms can better target temporal and spatial variations of thrombus. Moreover, different elastic micro- and nanomaterial response strategies (depolymerization, charge reversal, size alteration, shape change) can be designed to handle the dynamic changes in the thrombus microenvironment.

#### Nonpharmacological thrombolysis

**Fig. 12.**
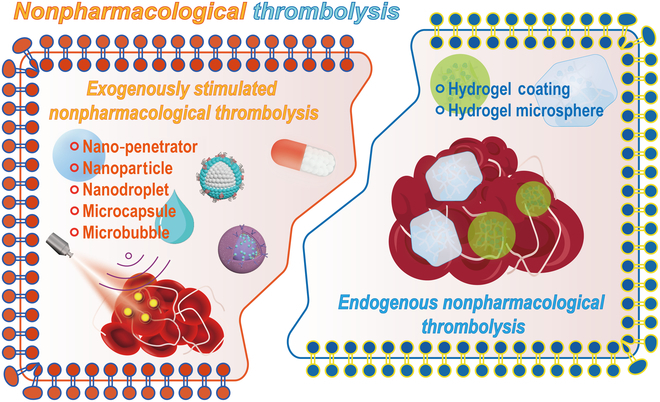
Schematic illustration of nonpharmacological thrombolysis based on different type of elastic micro-nano materials.

The development of thrombolytic drug delivery systems aims to overcome pharmacokinetic limitations, such as short half-life and poor targeting, which reduce efficacy and increase systemic risks. The current research focuses on developing advanced platforms to improve circulatory stability and site-specific delivery, enhancing therapeutic outcomes and safety. In addition, some thrombolytic drugs may lead to continuous inactivation of clotting factors, which results in delayed hemostatic recovery, further leading to bleeding and other serious life-threatening side effects [190,191]. Due to the potential side effects of thrombolytic drugs, research on nonpharmacological thrombolysis with elastic micro-nano materials is gradually emerging. As shown in Fig. 12, it mainly includes exogenous stimulatory nonpharmacological thrombolysis and endogenous nonpharmacological thrombolysis.

##### Exogenously stimulated nonpharmacological thrombolysis

**Fig. 13.**
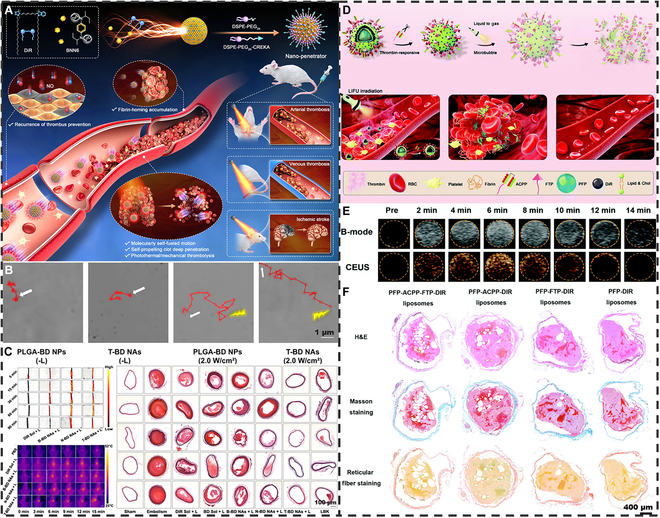
Exogenously stimulated nonpharmacological thrombolysis. (A) Schematic diagram of molecularly self-fueled nano-penetrator for nonpharmaceutical treatment of thrombotic diseases. (B) Movement trajectories of T-BD NAs and PLGA-BD NAs incubated in PBS (pH 7.4) at the same dose of DiR/BNN6 under laser irradiations (0 and 2 W/cm^2^, 60 s). (C) Intra-arterial thrombolysis in Sprague–Dawley (SD) rats. Reproduced with permission from [194]. Reprinted under the terms of the CC-BY 4.0. (D) Schematic illustration of the theragnostic functions of thrombin-responsive phase transition liposomes. (E) Ultrasound images of PFP-ACPP-FTP-DiR liposomes and ACPP-FTP-DiR liposomes under LIFU irradiation in B-mode and contrast-enhanced mode at different time points. (F) H&E, Masson, and reticular fiber staining images of inferior vena cava thrombosis in SD rats after 5-min treatment with the corresponding method. Reproduced with permission from [198]. Copyright 2020, The Royal Society of Chemistry.

Exogenous stimulated nonpharmacological thrombolysis is similar to drug delivery, mainly through NIR, ultrasound, and other exogenous stimuli targeting specific components of the thrombus microenvironment for targeted thrombolysis [192,193]. Photothermal induced nonpharmacological thrombolysis generally involves the release of large amounts of nitric oxide (NO) from NO donors by elastic micro-nano materials through NIR irradiation to generate bubbles. The subsequent cavitation effect causes both photothermal and mechanical thrombolysis. Zhang et al. [194] assembled a photosensitizer [1,1′-dioctadecyl-3,3,3′,3′-tetramethylindotricarbocyanine iodide (DiR)] with NO donors (BNN6) to form a self-propelled nano-armor-piercing projectile with high fuel loading and controllable motion characteristics (T-BD NAs) (Fig. 13A). The surface of this nanostructure was also modified with phospholipid PEG to enhance its in vivo circulation and attach CREKA peptide chains for thrombus targeting. After irradiation with a 2 W cm^−2^ 808-nm laser, T-BD NAs demonstrated good self-navigational and self-indicating thrombus-targeting accumulation, as well as excellent thrombolytic capability (Fig. 13B). In both arterial and venous thrombi, compared to free DiR, BNN6, unmodified B-BD NAs, NO-incapable N-BD NAs, and lumbrokinase (LBK), T-BD NAs had a stronger ability to inhibit thrombin activation and revascularization after 2 W cm^−2^ 808-nm NIR irradiation (Fig. 13C). Yang et al. [195] used an LBL self-assembly method to prepare an armor-piercing microcapsule (FGM@MC) encapsulating fucoidan, S-nitrosoglutathione, and melanin. FGM@MC induced by NIR can release large amounts of NO to generate bubbles, producing a cavitation effect to achieve mechanical thrombolysis. FGM@MC has a thrombolytic capacity comparable to that of UK, which does not interfere with the normal coagulation process and has a favorable biosafety profile. As previously mentioned in this review, ROS-mediated oxidative stress in the thrombus microenvironment is one of the factors to promote thrombus expansion. Zhang et al. [196] designed a surface-modified CREKA peptide chain encapsulated with Prussian blue (PB) and perfluoropentane (PFP) in PLGA nanodroplets (PB-PFP@PC). After systemic injection, CREKA’s affinity for fibrin PB-PFP@PC was able to target the thrombus site, and PB’s anti-inflammatory ability effectively eliminated ROS in the thrombus microenvironment and mitigated the effects brought about by inflammatory factors. In addition, the good photothermal conversion ability of PB can trigger the release of NO from PFP to achieve the effect of mechanical thrombolysis. The results showed that PB-PFP@PC was able to generate a mild photothermal of 45.8 °C at the thrombus site after 1.2 W cm^−2^ 808-nm NIR laser irradiation to promote NO production and was able to attenuate the level of inflammatory factors at the thrombus. Deng et al. [197] constructed a biomedium-driven nanoscavenger to obtain BNN6@PDA@CREKA (B@P@C) by loading NO donor (BNN6) onto hollow asymmetric PDA NPs modified by CREKA peptide chain. The PDA has excellent photothermal conversion capabilities, converting NIR light energy into thermal energy and transferring this thermal energy to BNN6. BNN6 releases NO bubbles for mechanical thrombolysis, and NO release also reduces platelet adhesion activity and vasodilatory capacity to prevent thrombus recurrence.

In addition to photothermal-induced nonpharmacological thrombolysis, US-induced nonpharmacological thrombolysis goes one step further by using the bubble and cavitation effect for mechanical thrombolysis. Yang et al. [198] reported a thrombin-responsive phase-change liposome that incorporates a liquid PFP core and is modified with activatable cell-penetrating peptides (ACPPs) and fibronectin-binding ligands (FTPs), which facilitate effective targeting and accumulation of liposomes within the thrombus (Fig. 13D). After targeting the thrombus fibrin, the liposome was able to perform the function of disrupting and penetrating the thrombus by acoustic droplet vaporization under low-intensity focused ultrasound (LIFU). As shown by thrombus photographs and hematoxylin and eosin (H&E) staining results in Fig. 13E, PFP-ACPP-FTP-DiR liposomes can ablate and penetrate thrombi compared to other controls (Fig. 13F). Zhong et al. [199] constructed a multi-functional phase-change thrombolytic nanomedicine by encapsulating PFH droplets in PLGA NPs and loading Fe_3_O_4_ NPs onto the NP surface, which were further modified to target CREKA peptides. When administered, NPs could accumulate in thrombus formation and the clot was degraded by LIFU irradiation.

Exogenously stimulated nonpharmacological thrombolysis can get rid of the conventional idea of drug delivery and physical–mechanical thrombolysis by heat, air bubbles, etc., generated in the thrombus microenvironment. However, similar to drug delivery with exogenous stimulation, exogenously stimulated nonpharmacological method application scenario is often limited by insufficient penetration depth. Therefore, the selection of good physical sensitizers and the differentiated treatment of different thrombi are also important factors to consider.

##### Endogenous nonpharmacological thrombolysis

**Fig. 14.**
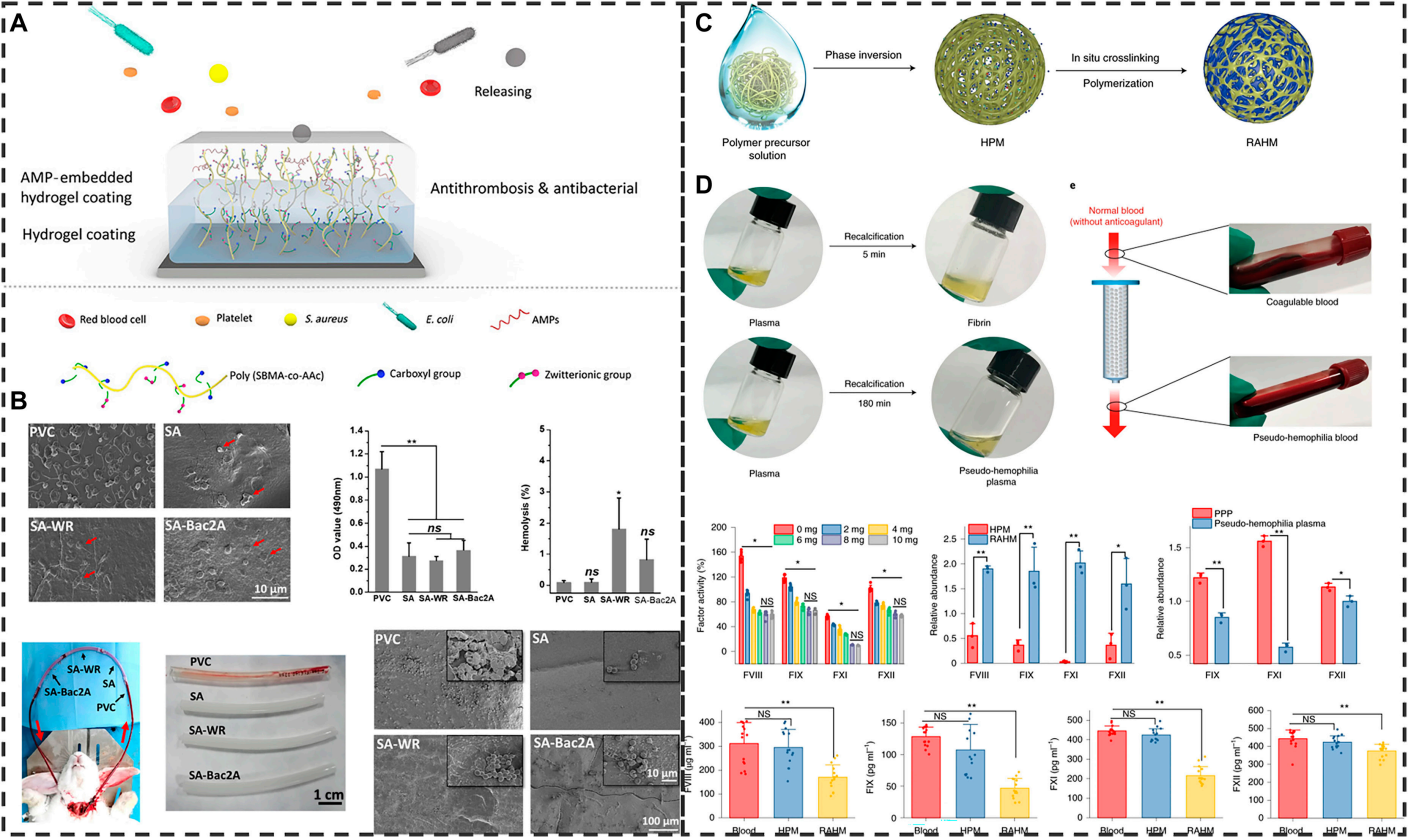
Endogenous nonpharmacological thrombolysis. (A) Proposed chemical structure of the adenosine monophosphate (AMP)-embedded hydrogel coating. (B) Anti-platelet adhesion ability of hydrogel coatings. Reproduced with permission from [203]. Copyright 2021, American Chemical Society. (C) Schematic of the preparation of the reinforced anticoagulant hydrogel microspheres (RAHMs). (D) Hydrogel microspheres adsorb clotting factors for blood-thinning purposes. Reproduced with permission from [204]. Copyright 2021 Springer Nature.

Research on nonpharmacological thrombolytic therapy is also gradually being established due to the side effects of conventional thrombolytic drug therapy on internal circulation and bleeding [200]. Common exogenous stimulatory thrombolytic therapy is often limited by problems such as depth of penetration and gas dependence. To overcome the obstacles posed by these problems, the development of endogenous nonpharmacological thrombolytic therapy is also the focus of current research [201]. In recent years, surface-engineered materials with anticoagulant properties have received high attention in the field of blood contact medical devices [202]. Liu et al. [203] designed a thrombolytic antimicrobial hydrogel (SA-Bac2A), which was formed by polymerizing methacrylenesulfone betaine and acrylic acid to form a hydrogel, and then embedded with the antimicrobial peptides WR (WRWRWR-NH_2_) and Bac2A (RLARVVIRVAR) (Fig. 14A). SA-Bac2A has not only good antimicrobial ability but also good thrombolytic ability. SA hydrogel had a good ability to inhibit platelet adhesion in a blood contact device. Semiquantitative analysis of platelet adhesion showed that SA hydrogel significantly reduced platelet adhesion compared to the original polyvinyl chloride (PVC) catheter. This blood-compatible and platelet adhesion-inhibiting hydrogel coating is expected to be used for antimicrobial functionalization of blood-related medical devices (Fig. 14B). Song et al. [204] introduced a new thrombolytic strategy for anticoagulation by designing a highly safe material capable of contacting blood in an extracorporeal circuit and providing transient local hemodilution (Fig. 14C). An enhanced anticoagulant hydrogel microsphere was prepared by infiltrating sulfonyl, hydroxyl, and carboxyl groups with blood-thinning ability into the hydrogel 3D lattice by electrospraying hydrophobic polyethersulfone with blood-purifying ability and hydrophilic N-vinyl-2-pyrrolidone as the backbone. The hydrogel microspheres demonstrated the ability to adsorb coagulation factors VIII, IX, and XI in both in vitro and beagle dog in vivo experiments, creating an environment that inhibited coagulation factor activity (8 to 30%) (Fig. 14D). Based on the description of this experiment, this functionalized hydrogel microsphere has a very promising application in nonpharmacological thrombolytic therapy.

Nonpharmacological and elastic micro-nano materials with anti-platelet adhesion and anticoagulant properties could address the shortcomings of therapeutic approaches such as thrombolytic drugs and exogenous stimulation. However, the thrombus-targeting properties and biosafety of such elastic micro-nano materials in clinically complex human environments still need to be further verified.

## Design Strategy for High-Efficacy Thrombolysis

**Fig. 15.**
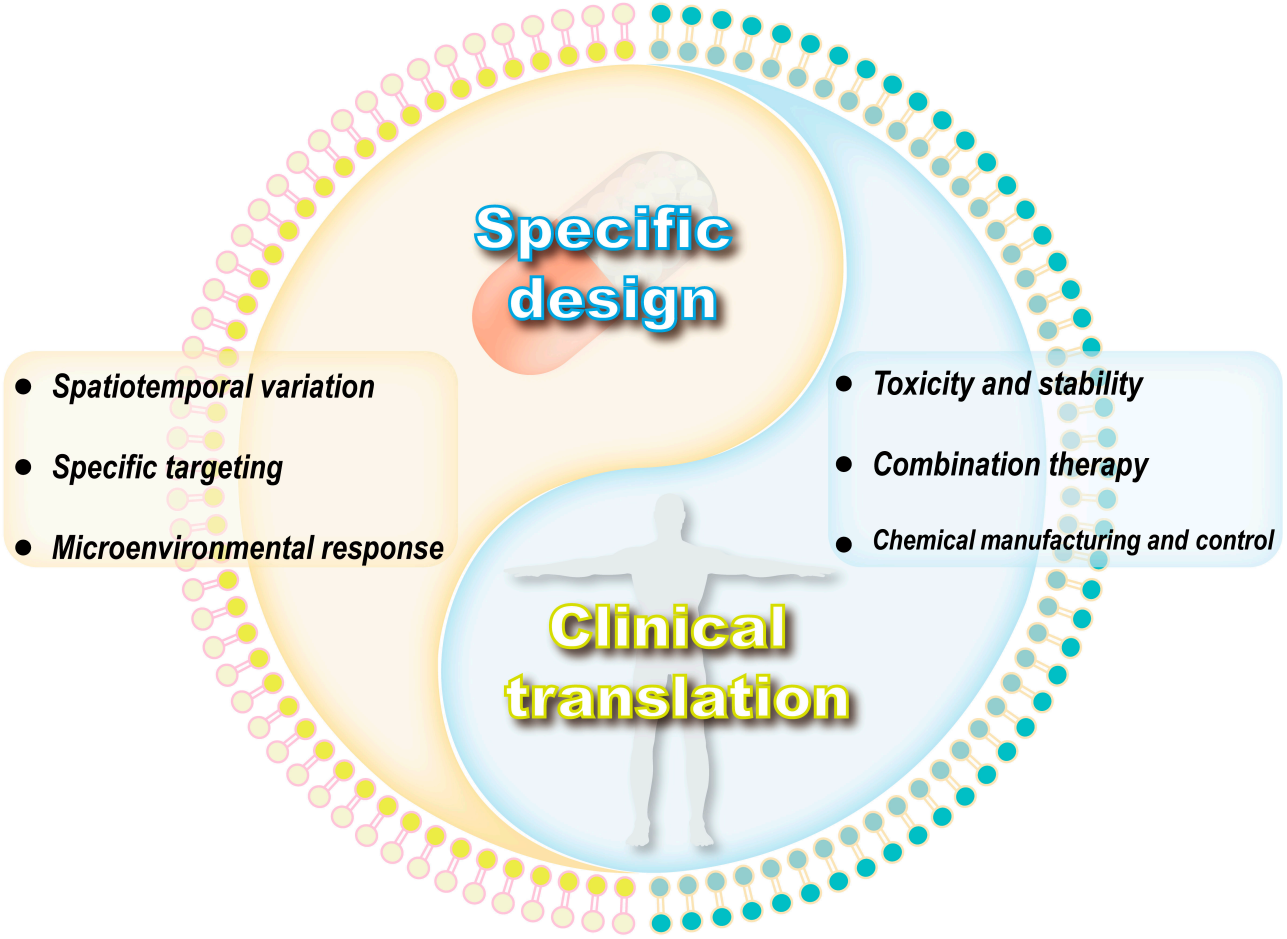
Schematic illustration of the design strategy for thrombus therapy of elastic micro-nano materials.

At present, elastic micro-nano materials have already appeared in thrombosis diagnosis and treatment due to their strong blood circulation and drug delivery capacity, but whether elastic micro-nano materials can play a proper role in the clinical human body still needs to solve a series of problems, such as specific design and clinical translation (Fig. 15).

### Specific design

The site of thrombosis determines the type of thrombosis, the period of thrombosis determines the status of biomolecules and biomechanics in the thrombus microenvironment, and the temporal and spatial variability of thrombosis determines the ease or difficulty of thrombosis treatment. The temporal and spatial variation of thrombus is one of the factors to be considered in the diagnosis and treatment of thrombus with elastic micro-nano materials. For example, white thrombus (arteries) is mainly composed of platelets and fibrin, so the pore size inside their clots is small. It is suitable for small-sized elastic micro-nano materials to penetrate and accumulate platelets and fibrin-targeted drug delivery systems. While red thrombus (vein) is mainly composed of fibrin and red blood cells, so the pore size inside its thrombus is larger [205]. It is suitable for the infiltration and accumulation of large-sized elastic micro-nano materials or the delivery system of fibrin-reducing and anticoagulant drugs.

Furthermore, different diagnostic and treatment strategies should be chosen for thrombus at different organ sites. The pathogenesis of thrombus in different organs is different, and diagnostic and treatment programs should be different for different symptoms. For example, cerebral thrombosis is a thrombus formed based on atherosclerosis and plaque formation in cerebral arteries. Under conditions of slow blood flow and low blood pressure, blood components adhere to the inner lining of the arteries [206,207]. Therefore, elastic micro-nano diagnostic materials that can penetrate the blood-brain barrier and promote the expansion of cerebral blood vessels are suitable. Pulmonary thrombosis is a disease caused by the obstruction of the pulmonary artery or its branches by blood clots from the venous system or the right heart. Its main clinical and pathophysiological characteristics are the dysfunction of pulmonary circulation and respiration [207,208]. It is more suitable for anticoagulant and defibrillator drugs such as warfarin and UK or elastic micro-nano diagnostic materials that can overcome the pulmonary mucosal barrier. There are also differences between different venous thromboses, which mainly include superficial veins located in subcutaneous fat and deep veins located in the muscular layer of the skeleton [209,210]. In patients with superficial vein thrombosis, a sudden acute inflammatory response occurs, resulting in a clot that firmly adheres to the vein wall and is not easily dislodged. Deep vein thrombosis tends to dislodge from the vein under muscular compression to form an embolus and cause an embolism. Therefore, superficial vein thrombosis is more suitable for thrombolytic elastic micro-nano materials with anti-inflammatory properties or inflammation-responsive drug delivery platforms, whereas deep vein thrombosis is suitable for targeted elastic micro-nano materials with higher efficacy.

Furthermore, different periods of thrombus development also require targeted research. For example, elastic micro-nano materials with anticoagulant activity or antiplatelet adhesion are more suitable for the early stages of thrombus development, before the formation of larger vascular emboli. Elastic micro-nano materials with anti-fibrin and shear stress-responsive properties are better suited for medium-sized thrombosis where the vessel width narrows. In the late thrombotic stage, when the vessel is completely blocked, elastic micro-nano materials with pH responsiveness and greater penetration and accumulation capacity are more suitable for thrombosis.

### Clinical translation

One of the major obstacles to the use of novel delivery system in clinical applications is its potential toxicity. In the current study, both drug-carrying and nonpharmacological thrombosis diagnosis and treatment of elastic micro-nano materials had good blood circulation in vivo. Therefore, the pharmacokinetics of elastic micro-nano materials needed further investigation than just validation of hemocompatibility. Elastic micro-nano materials should be systematically studied in major organs, such as physiological sections and immunohistochemistry. For metabolic organs such as the liver and kidney, elastic micro-nano materials with high circulatory capacity may place an additional load on them. Except for targeting the thrombus microenvironment, elastic micro-nano materials are also exposed to various physiological environments in body fluid circulation, and the biostability of the material is also an important factor to consider. When exposed to physiological environments, elastic micro-nano materials may undergo oxidation, agglomeration, and uncontrolled degradation, which will directly affect the effectiveness of the materials in thrombosis diagnosis and treatment. In addition, more specific studies of thrombosis with different temporal and spatial variations should be performed. For example, toxicity studies of elastic micro-nano materials on brain tissue and the nervous system need to be investigated in the diagnosis and treatment of cerebral thrombosis. Pulmonary thrombosis should be studied for its effects on alveolar tissue and the respiratory system. Complications are also frequent in thrombotic diseases, and elastomeric micro-nano materials for different thrombi need to take into account the mitigation and treatment of complications. Elastic micro-nano materials that are responsive to internal and external stimuli such as ROS, pH, urea, enzymes, shear stress, magnetism, light, sound, and temperature may be difficult to use in a general clinical setting. Their clinical translation may be hindered.

A key factor limiting the clinical translation of elastic micro-nano materials is their chemistry, manufacturing, and quality control. Any medical product requires strict quality control with a range of factors such as size, morphology, surface charge, and drug loading all needing to be tightly controlled at optimal levels. Based on the optimized product parameters, the manufacturer only needs to adjust certain production processes, such as temperature and pressure, to produce a product within the safe limits of the final formulation. Furthermore, elastic micro-nano materials that enter the market with greater therapeutic efficacy, lower toxicity, or lower price than conventional drugs, or that are prepared solely through nanomedicines, or that require lower administration frequency or a more convenient route of administration than conventional drugs, determine the potential market of the product.

In clinical thrombosis diagnosis and treatment, small-molecule and protein drugs, such as uPA, tPA, UK, warfarin, and rivaroxaban, are still the mainstream of research and application, occupying a large proportion. In contrast, micro-nano materials, such as NPs, hydrogels, and liposomes, have not yet entered the clinical trial stage. They are still in the exploratory and dormant period, pending further exploration of their potential. However, it is worth noting that some micro-nano materials have successfully entered the market of clinical oncology treatment, such as doxorubicin hydrochloride liposomes, bupivacaine liposomes, and paclitaxel liposomes. Their excellent performance in oncology treatment undoubtedly lights up the clinical research of micro-nano materials thrombosis, which is of great reference significance and worthy of an in-depth investigation and learning to emulate. Nevertheless, these liposomal drugs mostly act as basic “transportation carriers” in clinical applications, focusing on macroscopic therapeutic effects. The physicochemical properties of liposomal drugs, especially the elastic properties closely related to the adaptability and function of the biological environment, are still poorly studied. The intrinsic mechanisms and multiple influencing factors have not yet been deeply explored. In fact, elasticity and other physicochemical properties are like hidden manipulators that deeply regulate the interaction between micro-nano materials and the complex dynamic thrombotic microenvironment. With the help of cutting-edge technologies and innovative ideas, researchers can deepen their understanding of these key themes related to the clinical translation of micro- and nanomaterials, which can open a new chapter in the development of elastically engineered micro-nano materials, and promote the advancement of clinical thrombosis therapeutic strategies. In addition, a single design may not have the desired efficacy and may be more costly. Therefore, there is a need to design multi-targeted and combination therapies to treat diseases. In thrombosis therapy, elastic micro-nano materials with multiple targeting and synergistic effects can also be designed for more effective thrombosis therapy.

## Conclusion and Perspectives

In this review, we summarize the relevant applications of elastic micro-nano materials in thrombosis diagnosis and treatment. Specifically, the thrombus microenvironment (thrombosis mechanism, thrombus biomechanics, and thrombus classification), elastic micro-nano materials (definition, preparation, and biological effects), elastic micro-nano materials in thrombus diagnostics (in vitro and in vivo imaging diagnosis), elastic micro-nano materials in thrombus drug delivery (ligand-mediated drug delivery, biomimetic strategy drug delivery, microenvironment-responsive drug delivery, biomechanically responsive drug delivery, and exogenous stimulus-responsive drug delivery), as well as thrombus therapy (exogenous stimulatory and endogenous nonpharmacological treatments) have been introduced in detail. Elastic micro-nano materials have excellent fluid circulation capacity, long life, poor cell internalization efficiency, and better targeting capability. These properties contribute to the application of elastic dimensional nanomaterials in drug delivery and biomedical imaging, which could be more adaptable to the diagnosis and treatment of thrombosis. Elastic dimensional nanomaterials modified with different ligands for thrombosis diagnosis and therapy can respond to different thrombotic microenvironments and can effectively target thrombi and accumulate through penetration at the thrombus site. Elastic micro-nano materials have shown better results than conventional clinical materials and drugs in both imaging and therapy. In recent years, research on elastic micro-nano materials has made great progress in both drug delivery systems and disease treatment. Some of these materials such as liposome microspheres have been used in clinical diagnosis and treatment. However, as a disease characterized by spatial and temporal variations, thrombosis has some problems with conventional treatments. Elastic micro-nano materials may alleviate these problems but also create some new problems. For example, differences in the treatment of early and late thrombosis, differences in the microenvironment of thrombosis at different sites, and so on still limit the clinical translation of elastic micro-nano materials for thrombosis treatment. There are also specific designs, standardized preparation processes, and biosafety issues that require further study.

In summary, elastic micro-nano materials offer unprecedented opportunities in the field of thrombosis diagnosis and treatment. Elastic micro-nano materials may be a more effective and durable platform for thrombosis diagnosis than conventional thrombus imaging contrast agents and thrombolytic drugs. After addressing some of the potential specificity and safety issues, elastic micro-nano materials are expected to become alternative agents for clinical diagnosis and treatment of thrombus. Moreover, the combination of traditional drugs with novel elastic micro-nano materials will also provide a new opportunity for thrombus diagnosis and treatment. Continued advances in novel technology such as artificial intelligence-aided materials design will facilitate the development of flexible micro-nano materials for clinical diagnosis and treatment of thrombus in the future.
